# PPARγ Is Activated during Congenital Cytomegalovirus Infection and Inhibits Neuronogenesis from Human Neural Stem Cells

**DOI:** 10.1371/journal.ppat.1005547

**Published:** 2016-04-14

**Authors:** Maude Rolland, Xiaojun Li, Yann Sellier, Hélène Martin, Teresa Perez-Berezo, Benjamin Rauwel, Alexandra Benchoua, Bettina Bessières, Jacqueline Aziza, Nicolas Cenac, Minhua Luo, Charlotte Casper, Marc Peschanski, Daniel Gonzalez-Dunia, Marianne Leruez-Ville, Christian Davrinche, Stéphane Chavanas

**Affiliations:** 1 Centre de Physiopathologie Toulouse Purpan, INSERM UMR 1043, CNRS UMR 5282, Université Paul Sabatier, Toulouse, France; 2 Laboratory of Virology, Wuhan Institute of Virology, Chinese Academy of Sciences, Wuhan, China; 3 Hôpital Necker-Enfants Malades, Assistance Publique-Hôpitaux de Paris, Paris, France; 4 Université Paris Descartes, Sorbonne Paris Cité, Paris, France; 5 I-STEM, INSERM U861, AFM, Evry, France; 6 CECS, UEVE U861, Evry, France; 7 Département d'Anatomie Pathologique, IUCT-Oncopole, Toulouse, France; 8 Neonatal Unit, Children’s Hospital, Toulouse, France; University of Wisconsin-Madison, UNITED STATES

## Abstract

Congenital infection by human cytomegalovirus (HCMV) is a leading cause of permanent sequelae of the central nervous system, including sensorineural deafness, cerebral palsies or devastating neurodevelopmental abnormalities (0.1% of all births). To gain insight on the impact of HCMV on neuronal development, we used both neural stem cells from human embryonic stem cells (NSC) and brain sections from infected fetuses and investigated the outcomes of infection on Peroxisome Proliferator-Activated Receptor gamma (PPARγ), a transcription factor critical in the developing brain. We observed that HCMV infection dramatically impaired the rate of neuronogenesis and strongly increased PPARγ levels and activity. Consistent with these findings, levels of 9-hydroxyoctadecadienoic acid (9-HODE), a known PPARγ agonist, were significantly increased in infected NSCs. Likewise, exposure of uninfected NSCs to 9-HODE recapitulated the effect of infection on PPARγ activity. It also increased the rate of cells expressing the IE antigen in HCMV-infected NSCs. Further, we demonstrated that (1) pharmacological activation of ectopically expressed PPARγ was sufficient to induce impaired neuronogenesis of uninfected NSCs, (2) treatment of uninfected NSCs with 9-HODE impaired NSC differentiation and (3) treatment of HCMV-infected NSCs with the PPARγ inhibitor T0070907 restored a normal rate of differentiation. The role of PPARγ in the disease phenotype was strongly supported by the immunodetection of nuclear PPARγ in brain germinative zones of congenitally infected fetuses (N = 20), but not in control samples. Altogether, our findings reveal a key role for PPARγ in neurogenesis and in the pathophysiology of HCMV congenital infection. They also pave the way to the identification of PPARγ gene targets in the infected brain.

## Author Summary

Congenital infection by human cytomegalovirus (HCMV) might result in permanent neurological sequelae, including sensorineural deafness, cerebral palsies or devastating neurodevelopmental abnormalities. Infants with such sequelae represent about 0.1% of all live births (>5500 per year in the USA). Given the considerable health and societal burden, a better insight on disease pathogenesis is urgently needed to design new therapeutic or prognostic tools. Here, we studied the impact of HCMV on neuronal development, using human neural progenitors (NSC) as a disease model. In particular, we investigated the outcome of infection on Peroxisome Proliferator-Activated Receptor gamma (PPARγ, a key protein in the regulation of metabolism, inflammation and cell differentiation. We disclosed that HCMV infection strongly increases levels and activity of PPARγ in NSCs. In vitro experiments showed that PPARγ activity inhibits the differentiation of NSCs into neurons. We also found increased PPARγ expression in brains of in utero infected fetuses, but not in controls, suggesting that PPARγ is a key effector of HCMV infection also in vivo. Our study provides new insights on the pathogenesis of HCMV infection and paves the way to the discovery of PPARγ-related molecules secreted in the infected brain.

## Introduction

Congenital infection by human cytomegalovirus (HCMV) is a leading cause of permanent abnormalities of the central nervous system [1]. About 1% of newborns are congenitally infected with HCMV each year in the USA, as a result of either primary infection of a seronegative mother, or reinfection / viral reactivation in a seropositive mother during pregnancy. Ten percent of congenitally infected newborns are symptomatic at birth, and most of them (60–90%) display neurological sequelae [2]. Further, 10 to 15% of congenitally infected newborns that are asymptomatic at birth show neurological disorder with onset later in infancy [2]. The most severely affected fetuses or newborns show brain development abnormalities such as microcephaly, lissencephaly or polymicrogyria [2–4]. The most frequent permanent sequelae include mental and/or psychomotor disabilities, sensorineural hearing or vision loss, and/or spastic cerebral palsies. Overall, patients with permanent sequelae represent up to 0.1–0.2% of all live births (>5500 per year in the USA). The direct annual care costs for patients are estimated at $1-$2 billion in the USA [5]. No vaccine or reliable prognosis tools are available to date, except for ultrasound examination of macroscopic brain abnormalities. Considering the dramatic health and societal burden of congenital HCMV infection, it is clear that a better insight on its pathogenesis is urgently needed to provide new therapeutic and prognostic tools.

Human cytomegalovirus (HCMV) is a beta herpes virus that infects and replicates in a broad spectrum of organs and cell types. Infection of neural progenitor cells (NPCs) in the developing brain is thought to be a primary cause of the neurological sequelae due to HCMV congenital infection. Consistent with this hypothesis, studies using mouse brain slices or neurospheres reported that murine cytomegalovirus (MCMV) preferentially infected NPCs in the developing brain [6, 7]. Further studies by others and us showed that mouse or human NPCs obtained from neonatal autopsy tissues were permissive to HCMV infection in vivo or ex vivo [8–11]. These reports, however, revealed considerable diversity in the phenotype of NPCs following HCMV infection. Indeed, HCMV infection of neural progenitors was found to (i) inhibit self-renewal and proliferation, along with the induction of apoptosis [11], (ii) inhibit astrocyte differentiation [12], (iii) result in premature and abnormal differentiation [8], (iv) reduce the number of proliferating CD24-expressing NPCs [10]. Whatsoever, common to all studies was the observation that HCMV infection impaired the differentiation of NPC into neurons. Accordingly, two recent studies showed defective neuronal differentiation of neural stem cells generated from human induced pluripotent stem (iPS) cells upon in vitro HCMV infection [13, 14]. Despite these advances, the specific cellular and molecular mechanisms underlying the impaired neuronogenesis consecutive to HCMV infection still remain elusive. Given that a number of studies have established that peroxisome proliferator-activated receptor γ (PPARγ) is critical for proper brain development (reviewed in [15]), we reasoned that PPARγ may be involved in the impact of HCMV infection on neural progenitor cells. PPARγ is a ligand-dependent transcription factor, member of the nuclear receptor superfamily, which plays key roles in regulating cellular function and tissue homeostasis [16, 17]. Natural PPARγ ligands include 15-deoxy-∆^12,14^ prostaglandin (PG) J2 (15d-PGJ_2_), 15-hydroxyeicosatetraenoic acid (15-HETE), 9- or 13-hydroxyoctadecadienoic acid (9/13-HODE), all derived from oxidization cascades of poly-unsaturated fatty acids [16].

Here, we describe a new model of infection based on highly neuronogenic human neural stem cells (NSCs) derived from embryonic stem (ES) cells [18]. With this model, we examined the role of PPARγ in the neuropathophysiology of HCMV congenital infection. We further extended our observations to a collection of autopsy samples from HCMV-infected fetuses.

## Results

### NSCs from human embryonic stem cells are permissive to HCMV infection

**Fig 1:**
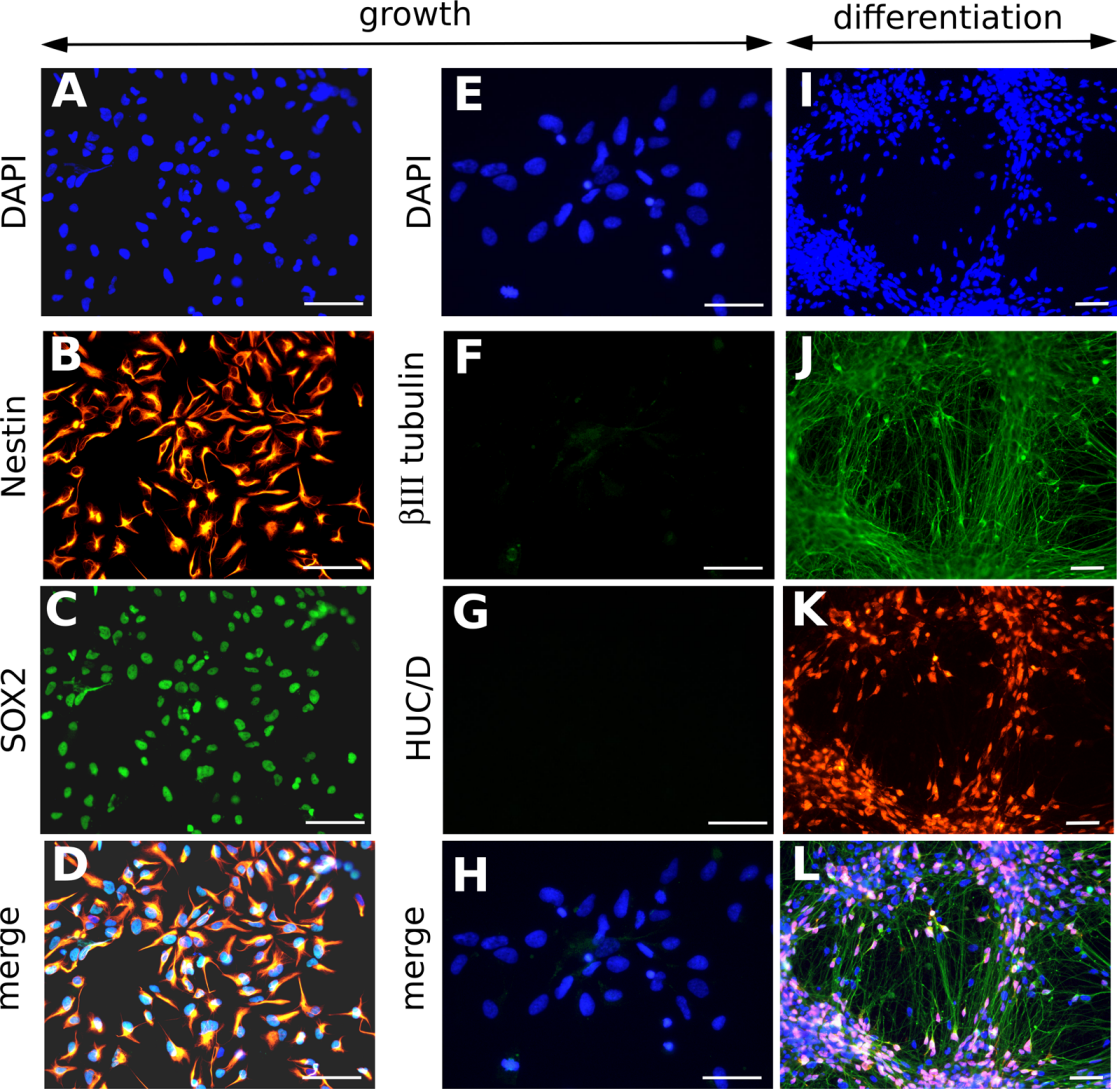
Characterization of neural stem cells from human ES cells (NSCs). Representative immunofluorescence analyses of NSCs cultured in growth medium (A-H) or in differentiation medium, 8 days after the onset of differentiation (I-L), using DAPI staining (A, E, I), or antibodies specific to Nestin (B), SOX2 (C), βIII tubulin (F, J) and HUC/D (G, K). Merged pictures are shown (D, H, L). In differentiation medium, neurons positive for βIII tubulin (J) and HUC/D (K) went alongside to undifferentiated NSCs, which nuclei appeared blue in the merged picture (L). Scale bar: 50 μm.

NSCs derived from embryonic stem cells showed self-renewal and continuous growth in defined conditions without the need of generating neurospheres. They expressed the multipotency marker SOX2 and the marker Nestin, and showed ability to differentiate into neurons positive for the markers HUC/D and βIII tubulin (Fig 1). An in-depth phenotypical characterization of NSC has been published elsewhere [19].

**Fig 2:**
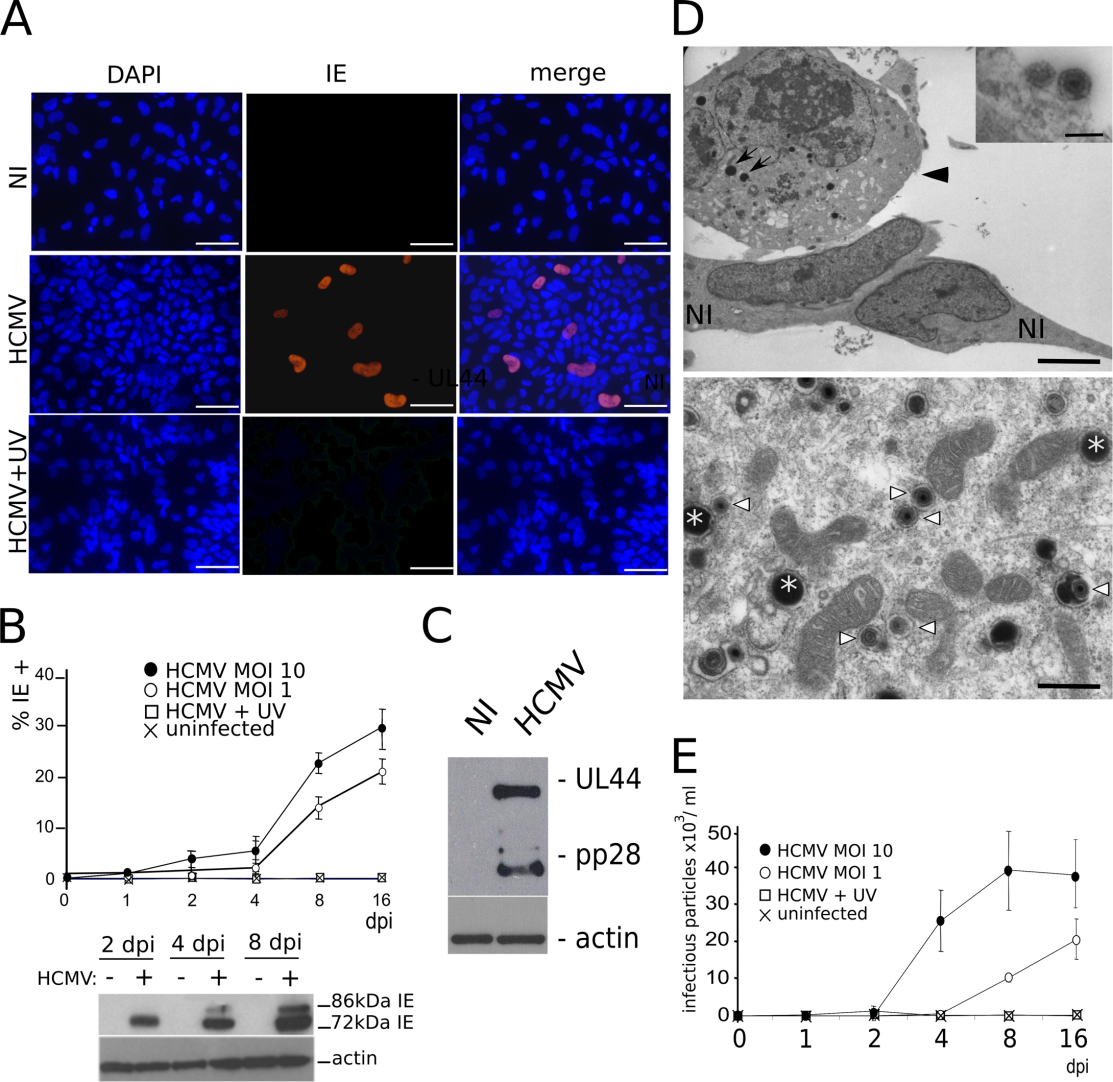
NSCs are permissive to HCMV infection. (A) Immunofluorescence analysis of NSCs infected by live (HCMV) or UV-irradiated (HCMV+UV) HCMV, or uninfected (NI), showing nuclear staining to the HCMV Immediate Early antigen (IE) two days post infection (dpi) at a multiplicity of infection (MOI) of 10. DAPI staining and merged pictures are shown. Scale bar: 50 μm. (B) Top: automated counting of immunofluorescence data showing increasing numbers of IE-positive NSCs over time in cultures infected by live HCMV at a MOI of 1 or 10, but not in cultures infected by UV-irradiated HCMV or in uninfected cultures. Data represent means ± CI of 2 independent experiments, each being performed in triplicate. Bottom: western blot analysis showing increasing levels along time of the 72 and 86 kDa isoforms of IE in infected NSCs (MOI 10). (C) Western blot analysis showing production of the early and late HCMV antigens UL44 and pp28, respectively, in infected NSCs (MOI 10), at 8 days pi. (D) Top: transmission electron microscopy of NSC cultures infected by HCMV (MOI 10), showing a cytomegalic NSC (arrowhead) and lipid vesicles (arrows), close to two morphologically normal NSCs (NI), and HCMV particles adsorbed onto the cell surface (inset). Scale bar: 5μm or 0.2 μm (inset). Bottom: transmission electron microscopy of the cytoplasm of an infected NSC, revealing mature viral particles (arrowheads) and dense bodies (asterisks). Pictures were taken 6 days after infection. Scale bar: 0.5μm. (E) Titration of viral particles present in the supernatants of infected NSCs (MOI 10). Supernatants were harvested at different times pi (horizontal axis) and were titrated on MRC5 fibroblasts. Data represent means ± CI of 2 independent experiments, each being performed in triplicate. Virus strain was AD169 except for panel A (VHL/E).

We first assessed the permissivity of NSC cultures to HCMV infection. Immunofluorescence analysis revealed that only few cells displayed a clear nuclear staining to HCMV Immediate Early antigen (IE) 24 h post infection (pi). At 48h pi, approximatively 5% of cells showed IE positive immunostaining (MOI 1 or 10) (Fig 2A). Thereafter, an increasing number of cells immunoreactive to IE were observed over time after HCMV infection, with up to 30% of IE-positive cells by 16 days pi (Fig 2B). Together, these results show that NSC cultures become progressively more permissive to HCMV infection overtime although the reason for this delayed kinetics is presently unknown. Our results are, however, consistent with previous reports showing that human neural progenitor cultures contain only 23% of IE-positive cells seven days after infection by the HCMV laboratory strain Towne when infected at a MOI of 1 [11]. All cells, including the IE-positive cells, remained immunoreactive to SOX2, suggesting that infection did not cause detectable changes in the stem cell status of NSCs (S1 Fig). As a control, no cell showed staining to IE when the inoculum had been previously UV-irradiated.

The 86-kDa form of IE, which is required for HCMV replication, was detected by western blot analysis as soon as 4 days pi (Fig 2B). Likewise, the early and late antigens UL44 and pp28 were immuno-detected from 8 days pi (Fig 2C). Electron microscopy revealed morphologically mature HCMV particles in the cytoplasm and pericellular space of infected NSCs, along with dense bodies and immature particles (Fig 2D). Titration of viral particles present in the medium of infected NSCs was performed using recipient MRC5 cells and fluorescence unit forming assay, which confirmed the presence of infectious HCMV particles (up to 4.10^4^/ml infectious particles released the 8^th^ day pi when the MOI was 10) (Fig 2E).

### HCMV infection impairs neuronogenesis

**Fig 3:**
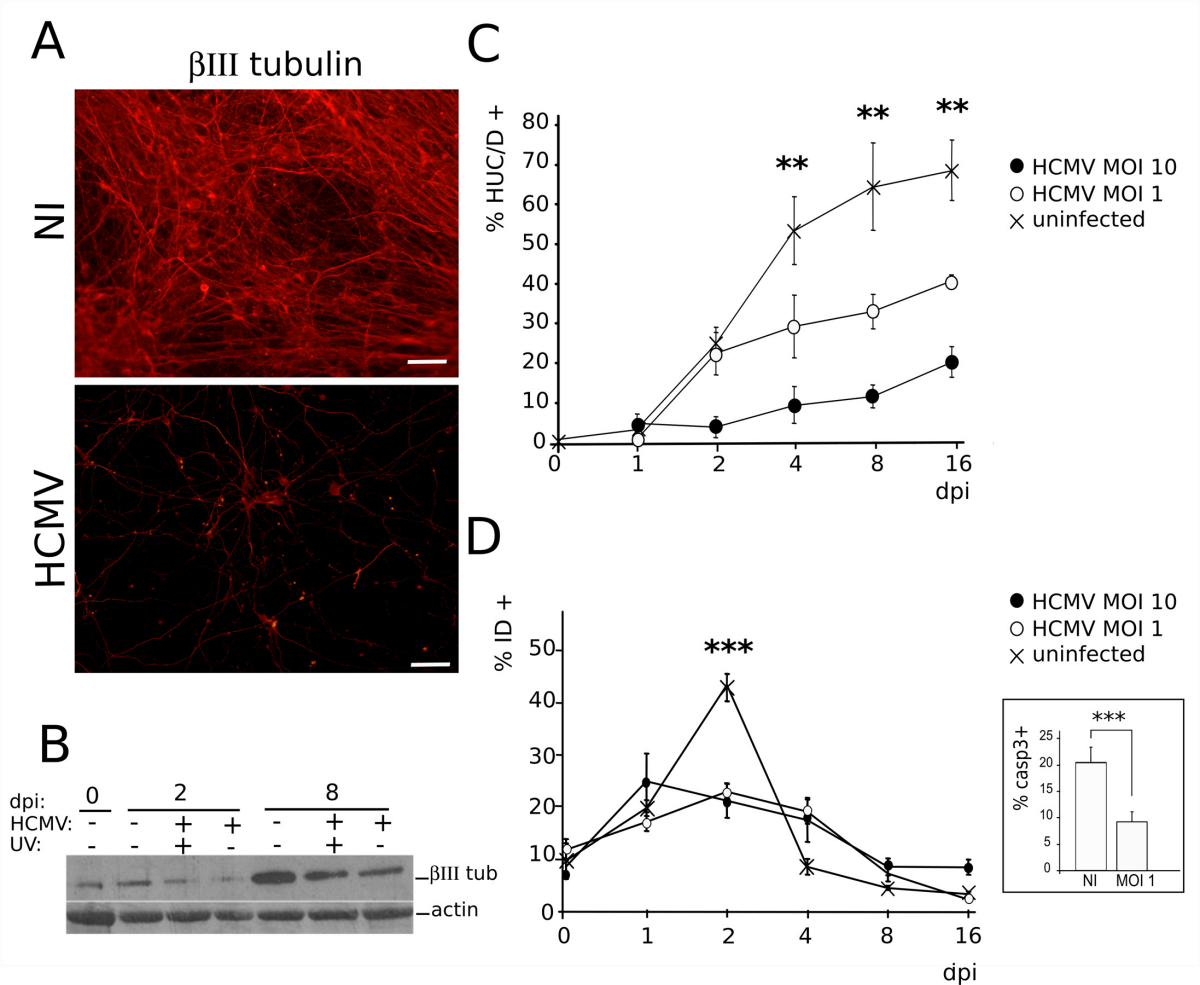
HCMV infection of NSCs impairs neuronogenic differentiation in vitro. (A) Representative immunofluorescence analysis of NSCs infected by HCMV at a MOI of 10 (HCMV) and uninfected (NI) NSC cultures, 6 days after the onset of differentiation (i.e., 5 days post infection [dpi]) using a βIII tubulin antibody. Scale bar: 50μm. (B) Western blot analysis of whole lysates from differentiating NSC infected by live HCMV at a MOI of 10 (HCMV) or by UV-irradiated HCMV (UV), or uninfected controls, showing decreased levels of βIII tubulin (βIII tub) in the infected cultures. (C) Automated immunofluorescence analysis of differentiating NSC cultures infected or not by HCMV with an HUC/D antibody (HUC/D+). Data represent means ± CI of 3 independent experiments, each being performed in triplicate. (D) Automated immunofluorescence analysis of differentiating NSC cultures infected or not by HCMV using the cell death marker Image-It Dead (ID). Inset: immunofluorescence analysis using an antibody specific to activated (cleaved) caspase 3 (casp3) of infected (MOI 1, 2 dpi) or uninfected (NI) NSC cultures. HCMV strain was AD169. Data represent means ± CI of 2 independent experiments, each being performed in triplicate. **: p<0.01, ***: p<0.005.

We next investigated whether infection altered the differentiation of NSCs into neurons. To initiate the differentiation of NSCs, it is critical to detach cells and to re-install them at a lower density on a fresh support, in the presence of increased concentration of laminin. In our preliminary experiments, we observed that the majority of infected cells were lost during these steps. As a result, differentiation of NSC cultures had to be initiated before any infection. Using this procedure, we observed that differentiating cultures of HCMV-infected NSCs displayed a dramatically decreased number of cells immunoreactive to βIII tubulin when compared to uninfected cultures (Fig 3A). Consistent with this observation, the overall level of βIII tubulin was strongly reduced in infected cultures (Fig 3B). To better appreciate the HCMV-triggered blockade of neuronal differentiation, we set up an automated procedure to screen populations of differentiating NSC grown in a 96-well plate format, based on the nuclear markers HUC/D (neurons) and SOX2 (NSCs). This analysis confirmed that the absolute and relative numbers of generated neurons decreased strongly and significantly as soon as 4 days pi in infected cultures (Fig 3C). At this stage, uninfected NSC cultures yielded 52% of neurons, whereas HCMV-infected NSCs generated 29% or 10% of neurons when infected, respectively, at MOI of 1 or 10 (p<0.008). At day 16 pi, uninfected NSC cultures yielded nearly 68% of neurons, while HCMV-infected NSCs generated 39% or 20% of neurons when infected, respectively, at MOI of 1 or 10 (p<0.01). Labeling for Ki67 antigen revealed no change in the proportion of dividing cells in the infected populations as compared to their uninfected counterparts, indicating that there was no concomitant increase in proliferative NSCs. Finally, we analyzed the cell death rate in infected or uninfected cultures. In control cultures, 44% of cells underwent developmental cell death within the two first days after onset of differentiation (Fig 3D). This event is classically observed during NSCs differentiation. Interestingly, HCMV infection appeared to limit this wave of developmental cell death, since the death rate was strongly and significantly decreased at day 2 pi in infected cell populations (22%; p<0.0032). This was confirmed by analyzing caspase 3 activation 48 h after infection, which revealed a significant decrease in the number of apoptotic cells among infected populations as compared to their uninfected counterparts (9% vs. 20%; p<0.0043, Mann-Whitney test)(Fig 3D).

### HCMV infection triggers PPARγ levels and activity in NSCs

**Fig 4:**
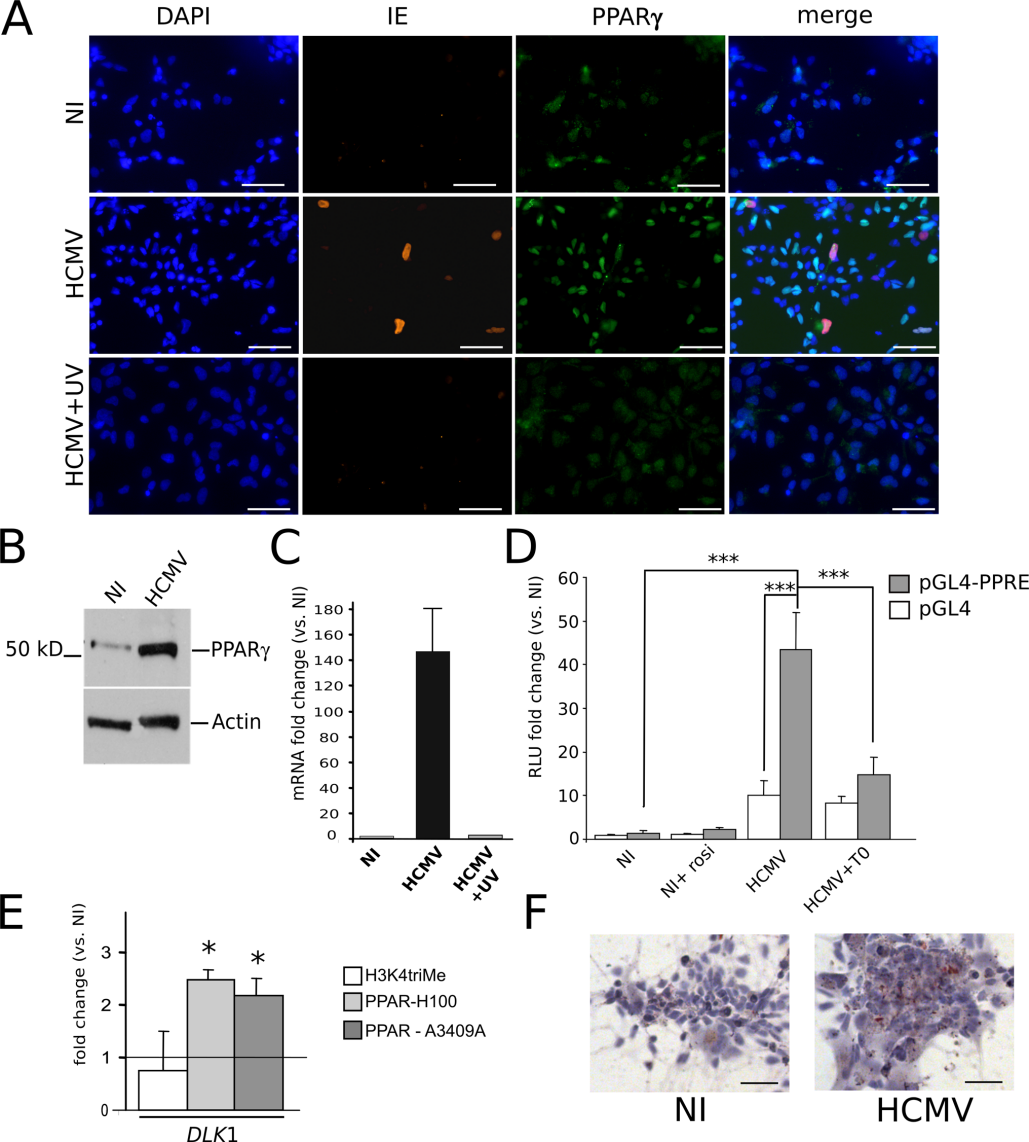
HCMV infection triggers the expression and activity of PPARγ in NSCs. (A) Immunofluorescence analysis using antibodies specific to IE or PPARγ showing strong nuclear staining of PPARγ in NSCs infected by HCMV at a MOI of 10 (HCMV), as compared to non infected NSC cultures (NI) or NSCs infected with UV-irradiated HCMV (HCMV+UV). In merged pictures, double stained nuclei appear cyan (PPARγ and DAPI) or magenta (IE and DAPI); nuclei stained by DAPI and PPARγ and IE antibodies appear purple. (B) Western blot analysis showing increased levels of PPARγ polypeptide in NSCs infected by HCMV (MOI 10) (HCMV) as compared to the uninfected control (NI), at 2 days post infection (dpi). (C) Q-RTPCR analysis showing increased levels of PPARγ transcript in NSCs infected by HCMV (MOI 10) (HCMV) as compared to the uninfected control (NI, value set to 1), or NSCs infected with UV-irradiated HCMV (HCMV+UV) at 2 dpi. Data represent means ± CI of 2 independent experiments, each being performed in triplicate. (D) Luciferase reporter assays showing non specific (pGL4) or PPARγ dependent (pGL4-PPRE) luciferase activity in uninfected NSCs (NI), uninfected NSCs treated with rosiglitazone (NI+rosi), NSCs infected by live HCMV at a MOI of 10, 2 days pi (HCMV), and NSCs infected in the presence of T0070907 (HCMV+T0). Data represent means ± CI of 3 independent experiments, each being performed in triplicate. (E) Chromatin immunoprecipitation assays using an antibody against K4-trimethylated histone 3 (H3K4triMe) as the positive control or two different antibodies against PPARγ (H100 and A3409A), showing increased occupancy by PPARγ of PPREs within the *DLK1* gene in NSCs infected by HCMV, as compared with uninfected NSCs. Shown are the fold change ratio from infected versus uninfected (NI) cells. Data represent means ± CI of 2 independent experiments, each being performed in triplicate. (F) Oil red O staining showing numerous lipid vesicles in infected NSC cultures (MOI 10) (HCMV) as compared to uninfected NSCs (NI). Virus strain was AD169. Scale bar: 50 μm. *: p<0.05; ***: p<0.005.

Uninfected NSCs displayed expression of only minute amounts of PPARγ, as shown by immunofluorescence (Fig 4A, top row) and western blot (Fig 4B) analyses. In contrast, immunofluorescence, western blot and quantitative mRNA analyses revealed high levels of PPARγ mRNA and protein in HCMV-infected NSCs (Fig 4A–4C). Interestingly, cells with positive PPARγ staining were much more numerous than IE-positive cells. The PPARγ staining was nuclear, suggesting that the receptor was in its active form (Fig 4A). This finding prompted us to investigate whether infection enhanced PPARγ transactivating activity. Activated PPARγ binds to cognate DNA sequences termed PPAR responsive elements (PPRE). We thus performed luciferase assays using a PPRE-containing, PPARγ-responsive, luciferase reporter plasmid (pGL4-PPRE-luc), and the corresponding control plasmid (pGL4). Stimulation of uninfected NSCs with rosiglitazone resulted in a small and non-significant increase in PPRE-luc activity (Fig 4D). Infection by live HCMV increased pGL4-driven luciferase activity (ten fold) suggesting a generalized enhanced transcriptional activity in infected NSCs (Fig 4D). More importantly, however, PPAR-specific luciferase activity as assessed by transfection with the pGL4-PPRE-luc reporter plasmid was strongly and significantly increased in infected NSCs (> 43 fold, p<0.0019) (Fig 4D). Incubation of infected NSCs with the specific PPARγ antagonist T0070907 induced a significant decrease of luciferase activity from PPRE-luc (p<0.0026) (Fig 4D), but no change in luciferase activity from the control plasmid pGL4-luc, further demonstrating specific increased PPARγ activity in infected NSCs. We next performed chromatin immunoprecipitation (ChIP) assays to examine the ability of PPARγ, or of whatever dimer containing it, to bind physically to cognate genetic sequences in infected NSCs. We used as a probe a genomic segment located in the 5’ promoter region of the *DLK1* gene, which binds PPARγ [20], and performed ChIP experiment with two different antibodies against PPARγ (Fig 4E). ChIP revealed a significant increase (> 2 fold, p<0.05) in the level of occupancy of the *DLK1* gene segment by PPARγ in infected NSCs. Last, Oil red O staining showed that infection was associated with the accumulation of lipid droplets in the cytoplasm of host NSCs, indicative of enhanced lipid metabolism and thus of PPARγ activity [21] (Fig 4F).

### Infected NSCs increase PPARγ expression levels in uninfected bystander cells

**Fig 5:**
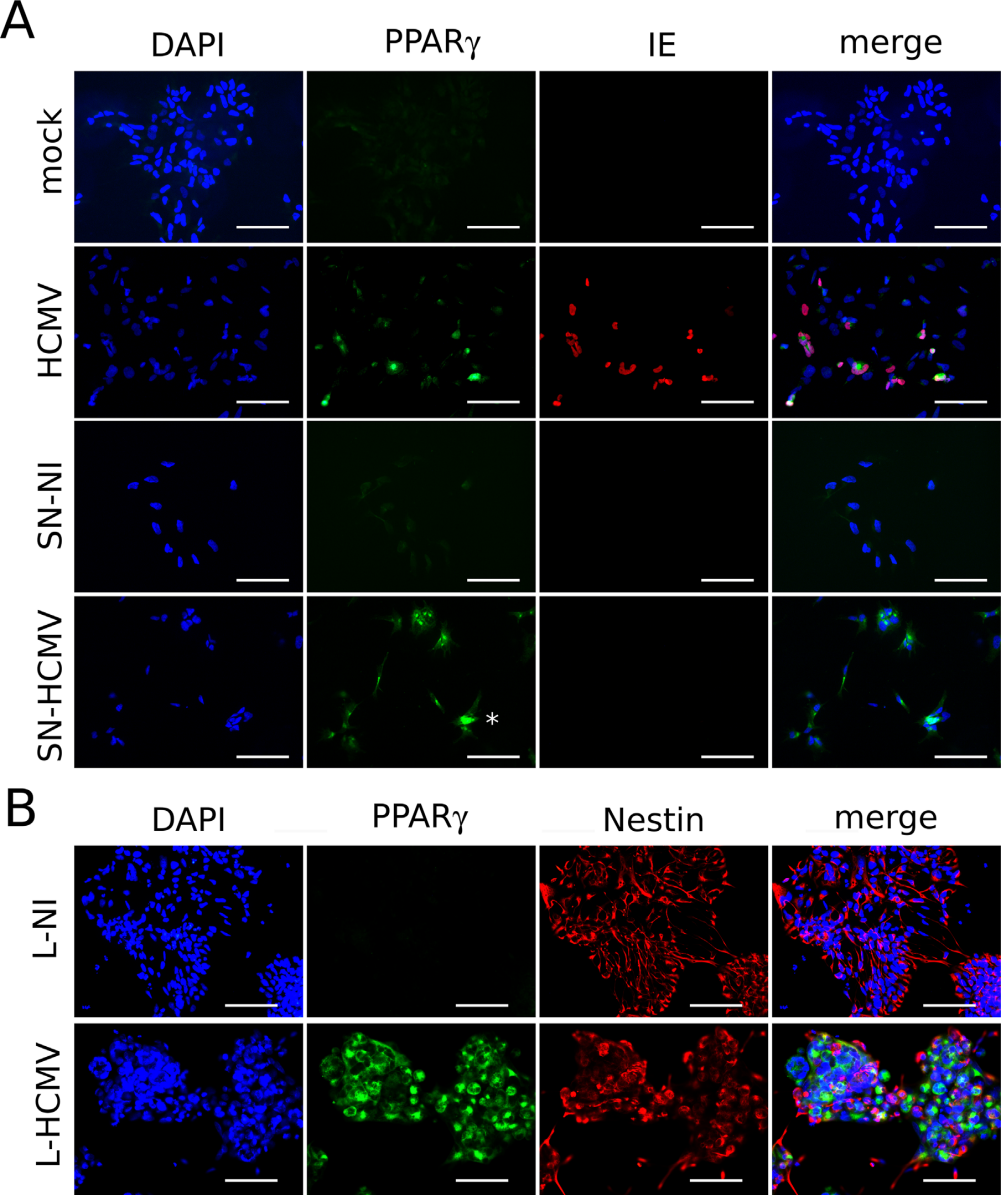
Soluble mediators from infected NSCs trigger the expression of PPARγ in uninfected NSCs. (A) Immunofluorescence analysis using antibodies specific to IE or PPARγ showing strong increase in PPARγ levels in NSCs infected by HCMV (HCMV), or in uninfected NSCs treated with supernatants prepared from HCMV-infected NSC cultures (SN-HCMV), as compared to uninfected NSCs treated with standard growth medium (mock), or NSCs treated with supernatants prepared from uninfected NSC cultures (SN-NI). The asterisk points to a representative cell with PPARγ nuclear staining. (B) Immunofluorescence analysis using antibodies specific to nestin or PPARγ showing strong increase in PPARγ levels in uninfected NSCs exposed to lipid extracts purified from the supernatants of HCMV-infected NSCs cultures (L-HCMV), as compared to NSCs exposed to lipid extracts purified from the supernatants of uninfected NSCs cultures (L-NI). Virus strain was AD169, and the MOI was 10 in all cases. Scale bar: 50 μm.

Since a large majority of NSCs showed increased PPARγ levels in infected cultures, even though they did not show IE expression, we explored the possibility that infected NSCs could exert a positive effect on PPARγ expression in the surrounding cells. To investigate whether infected NSCs could release soluble mediators able to trigger PPARγ expression, we purified the supernatants from infected or uninfected NSC cultures, 5 days post infection. We next treated uninfected NSCs with these supernatants (after an ultracentifugation step to eliminate viral particles) and we analyzed PPARγ expression two days after this medium change. NSCs treated with supernatants prepared from uninfected cells did not show any increase in PPARγ levels (Fig 5A). In contrast, NSCs treated with supernatants prepared from infected NSC cultures displayed markedly increased PPARγ levels, similar to HCMV-infected NSCs. Importantly, almost all cells of the monolayer appeared to be sensitive to exposure to the supernatant prepared from infected NSCs. As expected, no IE-positive cells were detected in the cultures treated with supernatants from infected NSCs, indicating efficient removal of virus particles during the ultracentrifugation step. These results thus show that infected NSCs release soluble mediators that contribute to increase PPARγ levels in uninfected bystander cells.

Because known PPARγ activators are polyunsaturated fatty acids [16], we next investigated the hypothetical role of lipids released by the infected NSCs. The total lipid fractions from the supernatants of infected or uninfected NSC cultures were purified by chromatography using C18 columns, followed by desiccation of the extract and solubilization in DMSO. Importantly, such a procedure is incompatible with virion survival, thereby preventing any effect due to virus carry-over. Next we added these lipid extracts to the culture medium of NSCs, at a final concentration of 0.1% (v/v). NSCs exposed to lipids purified from the supernatants of infected cultures displayed a strong increase in PPARγ levels, in sharp contrast to NSCs exposed to lipids purified from control uninfected culture supernatants (Fig 5B).

Our findings thus show that infected NSCs release soluble mediators able to activate PPARγ expression in uninfected NSCs, similar to direct infection per se, and that lipid components contribute to this bystander effect.

### Production of the PPARγ agonist 9-HODE is increased in infected NSCs

**Fig 6:**
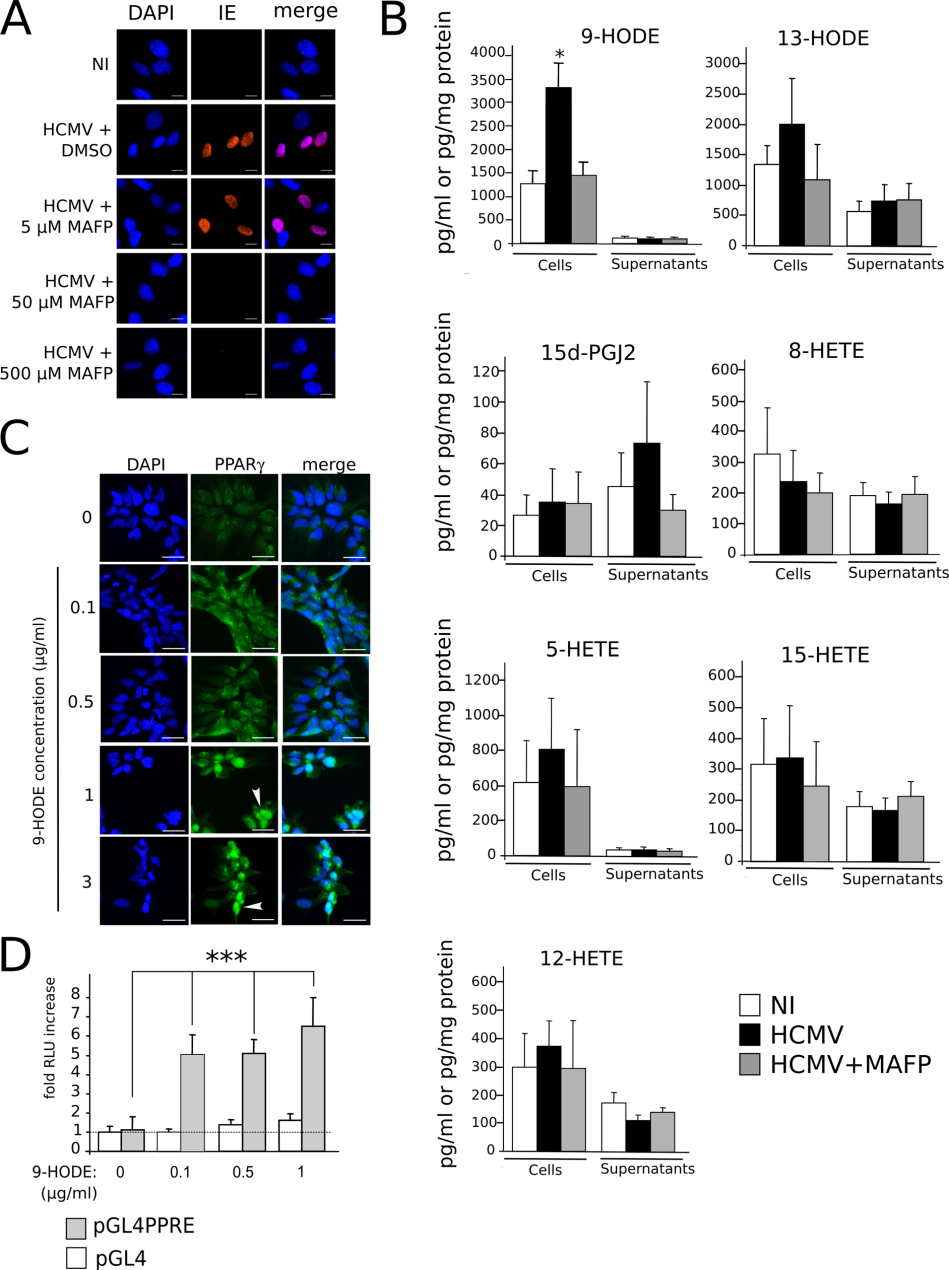
Increased production of PPARγ agonist 9-HODE in infected NSCs. (A) Immunofluorescence analysis of IE expression, showing that treatment of the HCMV inoculum by the PLA_2_ inhibitor MAFP impairs IE expression. NI: uninfected cells in medium containing 50 nM MAFP. (B) LC-MS/MS screening of PUFA-derived lipids produced in NSCs infected by live (HCMV) or MAFP-treated (HCMV+MAFP) HCMV, or in uninfected NSCs (NI), showing significant increase in 9-HODE levels in infected NSCs (top left). Amounts in supernatants are expressed in pg/ml, amounts in cell lysates are expressed in pg/mg protein. Data represent means ± CI of a minimum of 5 independent experiments, each being performed in triplicate. *: p<0.05. (C) Immunofluorescence analysis of PPARγ expression in uninfected NSCs showing increased PPARγ levels and nuclear translocation (arrowhead). (D) Luciferase assay showing increased PPARγ activity in NSCs stimulated by 9-HODE. HCMV strain was AD169. Data represent means ± CI of 2 independent experiments, each being performed in triplicate. ***: p<0.005.

We next investigated which ligands accounted for PPARγ activation during HCMV infection of NSC. Natural PPARγ ligands include 15-deoxy-∆^12,14^ prostaglandin (PG) J2 (15d-PGJ_2_), 15-hydroxyeicosatetraenoic acid (15-HETE), 9- or 13-hydroxyoctadecadienoic acid (9/13-HODE) [16]. The precursor of 15d-PGJ_2,_ PGD2, as well as 15-HETE and 9/13-HODE are generated by oxidization of arachidonic acid (AA) or linoleic acid (LA) by cyclooxygenase (COX) or 5/15- lipoxygenase (LOX). Release of AA or LA from membrane glycerophospholipids is catalytically driven by calcium-dependent phospholipase A_2_ (cPLA_2_) activity [22]. Interestingly, during HCMV virion assembly, cellular cPLA2 is packaged into the viral particle and remains within the tegument of the virions, as an “onboarded” cell-derived cPLA2 which is required for infectivity [23]. It has been shown that this cell-derived cPLA2 can be inhibited by treatment of the viral inoculum by the specific cPLA_2_ inhibitor methyl arachidonyl fluorophosphonate (MAFP) before infection [23, 24]. Accordingly, we observed that treatment of the inoculum by 50 μM MAFP abolished IE expression in NSCs (Fig 6A). This suggested a role for this virion-packaged cPLA_2_ in the biosynthesis of possible PPARγ activators derived from polyunsaturated fatty acids (PUFA). To test this hypothesis, we measured levels of candidate PUFA-derived PPARγ agonists in control NSCs, HCMV-infected NSCs and, as a control, NSCs infected by MAFP-inactivated HCMV. We used a novel, rapid and sensitive method based on high performance liquid chromatography coupled to tandem mass spectrometry (LC-MS/MS) [25] using lysates and conditioned culture media collected at 24 h pi (Fig 6B). Candidate PPARγ agonists were 9/13-HODE, 15-HETE, and 15d-PGJ_2_. We also investigated the amounts of 5/8/12-HETE, although they have not been formally identified as PPARγ agonists to date. No significant changes were detected in amounts of 15d-PGJ2, 13-HODE or 5/8/12/15-HETE in infected NSCs or the corresponding culture supernatants (Fig 6B). In contrast, levels of 9-HODE were significantly increased in lysates from HCMV-infected NSCs (> 2.4 fold; p<0.029) (Fig 6B). In uninfected control cell lysates, 9-HODE amounts were detected at 1440.3 pg (4.9 pmol) per mg of total cellular protein, whereas they rose to 3539.8 pg (12 pmol) per mg of protein in infected NSCs. Treatment of HCMV particles with MAFP prior to infection abolished this increase in 9-HODE amounts (Fig 6B), indicating that active virion-packaged cPLA_2_ is needed for efficient 9-HODE biosynthesis. Importantly, only low amounts of 9-HODE were found in the conditioned culture supernatants (approximately 0.1 μg/ml, i.e., 0.3 nM) and no difference was observed in supernatants between infected and control NSCs. We assume that this was likely due to poor stability of 9-HODE in the serum-free medium and/or high cell permeability to 9-HODE resulting in poor abundance in the medium. In any event, our results suggest that HCMV triggers 9-HODE biosynthesis, at least at early stages of infection. Next, we investigated the outcomes of 9-HODE on PPARγ activity in NSCs, independently from the infectious context. We first carried out immunofluorescence analysis using NSCs stimulated during 24h by a range of 9-HODE concentrations. This analysis revealed a dose-dependent increase in levels of PPARγ staining in NSCs in response to 9-HODE exposure (Fig 6C). We also evidenced the nuclear translocation of PPARγ in cells stimulated with 9-HODE at concentrations greater than 0.5 μg/ml (Fig 6C). We further investigated the effect of 9-HODE on PPARγ activity by using the more sensitive luciferase reporter assay, which showed significantly increased PPARγ activity in NSCs stimulated by 9-HODE from 0.1 μg/ml (p< 0.0022) (Fig 6D). Altogether, our results indicate that 9-HODE efficiently activates PPARγ in NSCs, even outside of the infectious context.

**Fig 7:**
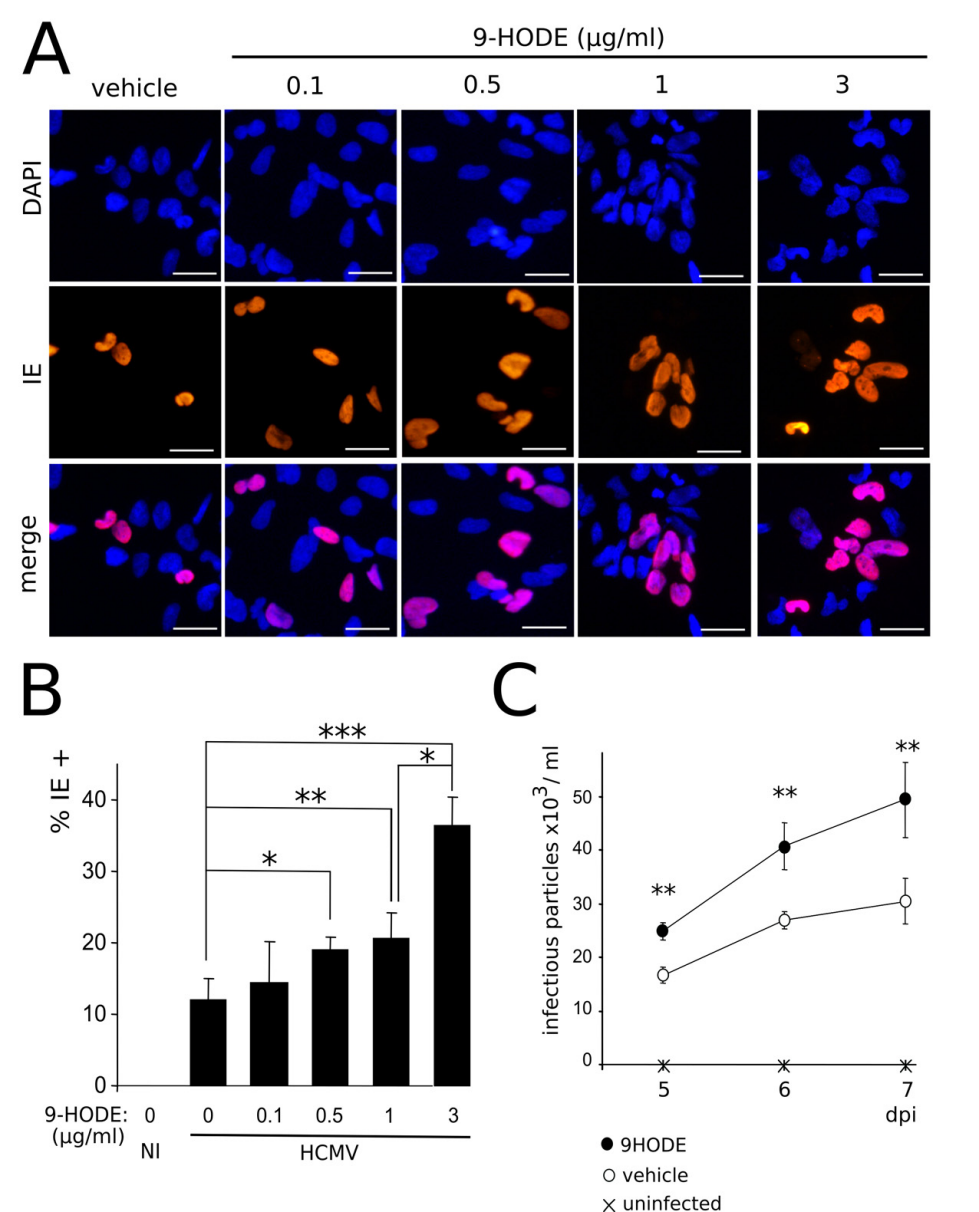
HCMV replication is enhanced by 9-HODE treatment. (A) Representative immunofluorescence results of IE expression in NSCs infected by HCMV at a MOI of 10 in the presence of increasing concentrations of 9-HODE or the vehicle, 48 h pi. (B) Immunofluorescence analysis showing increasing number over time of IE positive NSCs in cultures infected by live HCMV at a MOI of 10 in the presence of increasing concentrations of 9-HODE or the vehicle, 48 h pi. Data represent means ± CI of 2 independent experiments, each being performed in duplicate. NI: uninfected control. (C) Titration of the viral particles in supernatants of MRC5 fibroblast cultures treated beforehand with supernatants of NSCs infected by live HCMV at a MOI of 10 in the presence of 1 μg/ml 9-HODE or the vehicle, 48 h pi, or uninfected NSCs. HCMV strain was AD169. Data represent means ± CI of 2 independent experiments, each being performed in triplicate. *: p< 0.05; **: p< 0.01.

Since PPARγ enhances IE1/2 gene transcription and HCMV replication [26], these results prompted us to investigate the impact of 9-HODE on HCMV replication in NSCs. To this aim, we infected NSCs at a MOI of 10 in the presence of 9-HODE in the medium. Immunofluorescence analysis revealed that treatment with 9-HODE resulted in significantly increased amounts of cells immunoreactive to an antibody specific to IE, at concentrations from 0.5 μg/ml (p< 0.0043, Mann-Whitney test) (Fig 7A and 7B). Lastly, we carried out an HCMV titration assay. Culture supernatants from infected NSCs stimulated or not by 9-HODE were harvested at 5, 6 and 7 days pi, and were added to the culture medium of MRC5 fibroblasts. The day after, immunofluorescence analysis was performed to assess the number of cells immunoreactive to IE. Titration assay showed that MRC5 cultures incubated with supernatants from NSCs infected in the presence of 9-HODE contained significantly greater number of IE-positive cells (Fig 7C) (p<0.01, Wilcoxon test). Together, these findings show that HCMV replication is more efficient when stimulated by 9-HODE, consistent with the fact that HCMV uses PPARγ for its replication [26].

### PPARγ activation inhibits NSC neuronal differentiation

**Fig 8:**
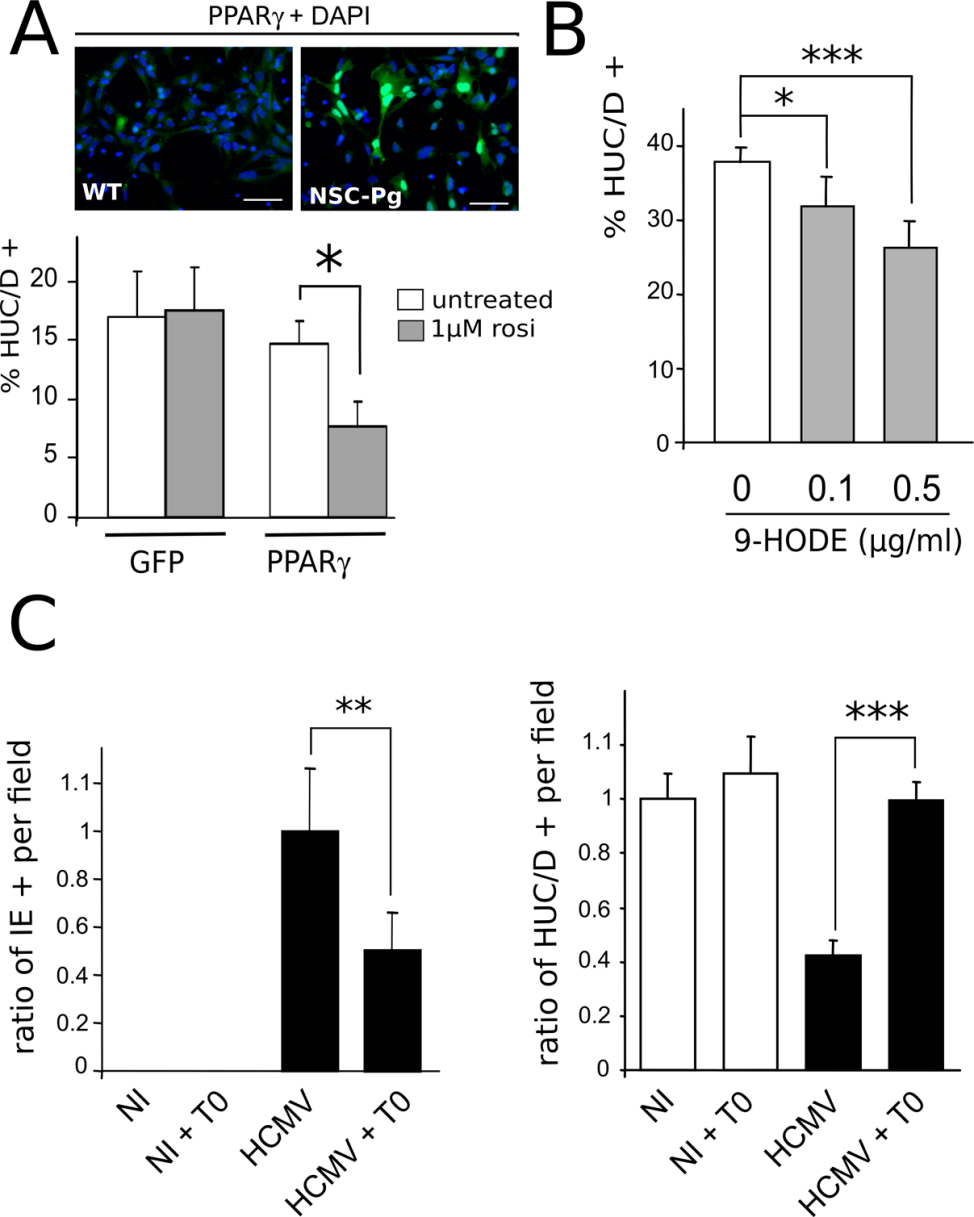
PPARγ activity negatively regulates neuronogenesis from NSCs. (A) Top: Representative immunofluorescence analysis of uninfected NSCs stably expressing PPARγ (NSC-Pg), using an antibody specific to PPARγ (green) (right). Wild type uninfected NSC cultures (WT) stained with the same PPARγ antibody (green) are shown as a control (left). Scale bar: 50 μm. Bottom: In vitro neurogenesis assay showing significantly decreased number of HUC/D positive neurons generated from NSC-Pg (PPAR) stimulated by 1 μM rosiglitazone as compared to either untreated NSC-Pg or NSC-GFP (GFP) stimulated or not by rosiglitazone. Data represent means ± CI of 2 independent experiments, each being performed in duplicate. (B) In vitro neurogenesis assay showing significantly decreased number of HUC/D positive (HUC/D+) neurons generated from wild type NSC treated by 0.1μg/ml or 0.5 μg/ml 9-HODE, as compared to control NSCs. Data represent means ± CI of 2 independent experiments, each being performed in duplicate. (C) Left: Analysis of the number of IE positive cells in NSCs infected or not by HCMV at a MOI of 1 and cultured in the presence of 10 nM T0070907 (T0) or the vehicle, 4 days pi, showing significantly lower number of IE positive NSCs in cultures with T0. Data are expressed as the ratio relative to the number of IE-positive cells found in untreated, infected NSCs, which was arbitrarily set to 1. Right: In vitro neurogenesis assay showing significantly increased number of HUC/D positive neurons generated from wild type NSC infected by HCMV at a MOI of 1 and treated by 10 nM T0070907, as compared to untreated, infected NSCs. Data are expressed as the ratio relative to the number of HUC/D-positive neurons found in untreated, uninfected NSCs, which was arbitrarily set to 1. Data represent means ± CI of 2 independent experiments, each being performed in duplicate. Virus strain was AD169. *: p< 0.05; **: p< 0.01; ***: p< 0.005.

We next investigated whether increased PPARγ activity could play a causative role in the defective neuronogenic differentiation of infected NSCs (Fig 8). First, we generated stably transduced NSCs that constitutively and strongly expressed either mouse PPARγ (NSC-Pg) or eGFP (NSC-GFP) (Fig 8A, top). Next, we carried out in vitro neuronogenesis assays to investigate the level of differentiation of such recombinant NSCs, stimulated or not by the PPARγ activator rosiglitazone. After seven days of differentiation, we observed that unstimulated NSC-GFP and NSC-Pg both yielded a relatively lower number of neurons (<20%) as compared to wild type NSC cultures, probably because of higher cell passaging, transduction and/or selection (Fig 8A, bottom). No significant difference was observed in the number of neurons generated in NSC-GFP cultures stimulated by the PPARγ activator rosiglitazone as compared to unstimulated NSC-GFP (Fig 8A, bottom). In contrast, a significantly decreased number of neurons was generated by NSC-Pg upon rosiglitazone stimulation (p<0.0006, Man-Whitney test) (Fig 8A, bottom). We next used wild type NSCs to investigate the impact of the activation of endogenous PPARγ in on neuronal differentiation in vitro. We used 9-HODE as the activator because, unlike rosiglitazone, it is able to increase both expression and activity of endogenous PPARγ in NSCs (Fig 6). We examined the number of HUC/D positive (HUC/D+) neurons generated from NSCs grown in the presence of 9-HODE at 0.1 μg/ml or 0.5 μg/ml, or in the presence of the vehicle (ethanol), after seven days of differentiation. 9-HODE stimulation at concentrations greater than 0.5 μg/ml resulted in strong cytotoxicity within this time period. In the absence of 9-HODE, NSC differentiation yielded 38% HUC/D positive neurons (Fig 8B). Significantly lower rates of differentiation were found in NSCs cultured in the presence of 9-HODE at a concentration of 0.1 μg/ml (31%; p<0.017) or 0.5 μg/ml (26%; p<0.002) (Fig 8B). Together, these findings establish that activated PPARγ is sufficient to impair neuronal differentiation of NSCs, even without infection. Lastly, we investigated whether pharmacological inhibition of PPARγ could improve neuronal differentiation in vitro from HCMV-infected NSCs. We carried out neuronogenesis assays with NSC infected by HCMV at a MOI of 1, in the presence of T0070907, a PPARγ-specific inhibitor. Because of the cytotoxicity of T0070907 on differentiating NSCs, we had to culture them on glass coverslips for no longer than 5 days, before examination of a randomly-selected set of optical fields (N = 12) and statistical analysis. When T0070907 was added to the culture medium 3 h pi, it strongly and significantly limited HCMV infection. Indeed, immunofluorescence analysis showed that T0070907-treated NSC populations contained two times less IE immunoreactive cells per field than untreated cultures, at 4 days pi (p<0.008, Mann-Whitney test) (Fig 8C, left). Indeed, untreated NSC cultures contained an average of 11% IE-positive cells (with a 5% confidence interval of 2.9%), whereas the T0070907-treated NSC populations contained an average of 5% of IE-positive cells (with a 5% confidence interval of 1.8%), consistent with our previous observations. This result, consistent with previous studies [26], also confirmed that PPARγ was efficiently inhibited by T0070907-treatment. As expected, infected NSCs showed defective differentiation and generated almost two times less HUC/D + neurons than uninfected cells (p<0.0014) (Fig 8C, right). Untreated NSC cultures contained from 20% to 40% of neurons per field among the total cell population. Also, uninfected NSCs differentiating in the presence of T0070907 showed no significant change in the abundance of neurons generated as compared to the untreated controls (Fig 8C). In contrast, infected NSCs differentiating in the presence of T0070907 yielded the same number of neurons as compared to the uninfected controls (Fig 8C). These results suggest that treatment by the PPARγ inhibitor T0070907 can reverse the effects of infection on neuronogenic differentiation in vitro, either directly through PPARγ inhibition, or indirectly through inhibition of viral replication resulting from PPARγ inhibition.

### PPARγ expression is increased in congenitally HCMV-infected fetuses

To assess the pathophysiological relevance of our experiments using NSCs, we next investigated the expression of PPARγ in fetal brain samples from aborted fetuses with congenital HCMV infection (N = 20) or from control subjects (N = 4). The clinical and pathological features are summarized in Table 1.

**Table 1:** Clinical and histopathological summary.

	Reference	GA (weeks)	Gender	Weight (g)	IUGR	μC	μG	PμG	LC	VMG	EPT	CAL	other	IHC (IE+)
Patients	**1100117**	23	M	800				+		+	+			**9**
	**4752**	23	M	520		+	+	+	+	+		+ ^v^		**36**
	**3955**	23	M	410	+	+		+		+			PVLM	**180**
	**1000239**	23	F	530			+				+			**3**
	**4261**	23	F	630					+					**8**
	**4099**	23	F	430			+	+	+					**220**
	**900188**	24	M	527		+	+	+		+				**23**
	**4688**	24	M	620		+	+					+ ^gz, v^		**59**
	**4350**	24	F	800										**1**
	**4135**	24	F	770			+		+					**9**
	**1100508**	25	M	780			+			+				**72**
	**3918**	25	F	615	+	+						+ ^v, c, wm^		**47**
	**1000207**	25	M	700			+			+		+ ^bg^		**3**
	**1300547**	27	F	815	+	+	+	+		+	+			**301**
	**4639**	27	M	1150			+		+	+	+			**8**
	**4543**	27	M	1170										**6**
	**1100328**	27	M	1050	+		+	+		+	+			**25**
	**4082**	28	F	1150								+ ^wm, bg, cn^		**56**
	**1100075**	28	M	1020			+							**5**
	**4460**	28	F	1150		+	+							**38**
Controls	**P1200496** ^1^	23	F	470										**0**
	**P1200094** ^2^	23	F	545										**0**
	**P1200401** ^3^	25	M	820										**0**
	**P1200243** ^4^	28	F	580	+									**0**

GA: gestational age based on last menstrual period, IUGR: intra uterine growth retardation, μC: microcephalia, μG: microgyria, PμG: polyesmicrogyria, LC: lissencephaly, VMG: ventriculomegaly, EPT: ependydimitis, CAL: calcifications, PVLM: periventricular leukomalacia, IHC: immunohistochemistry. IHC indicates the total number of IE immunoreactive cells detected per 8-μm brain section. Locations of calcifications were: (^v^) ventricles, (^gz^) germinative zone, (^c^) cortex, (^wm^) white matter, (^bg^) basal ganglia, (^cn^) caudate nucleus. Causes of abortion of control subjects were: (^1^) cardiopathy and DiGeorge syndrome, (^2^) premature rupture of membranes, anamnios and chorioamniotitis, (^3^) renal failure, (^4^) atrioventricular canal and omphalocele.

**Fig 9:**
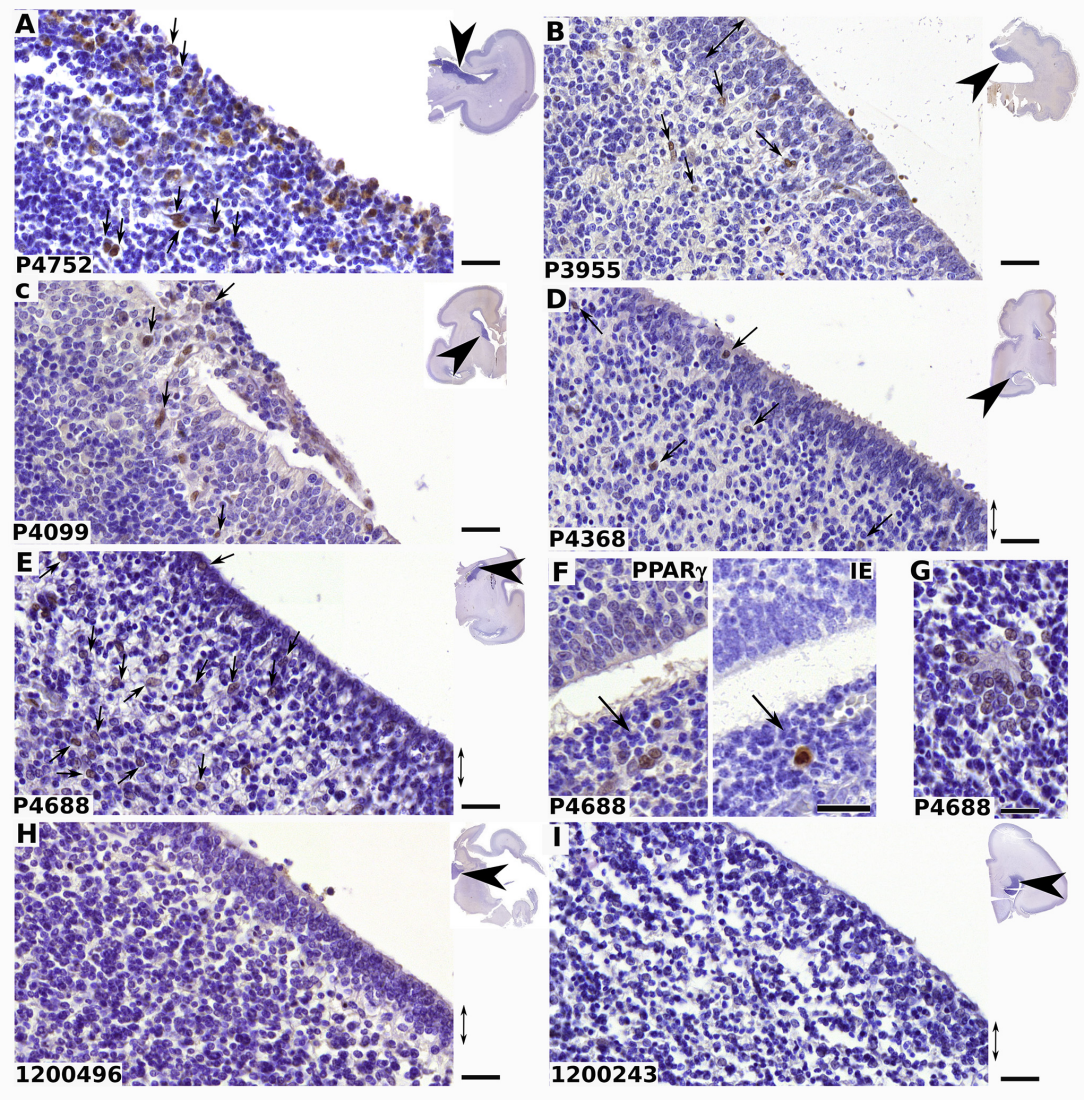
Nuclear PPARγ expression in germinative zone of HMV-infected human fetal brains. Shown are representative results of immunohistological staining of brain sections from fetuses infected by HCMV (A-G) or from controls (H, I) using antibodies against PPARγ (A-E; F, left; G-I) or IE (F, right). The reference number of each donor is indicated at the bottom left of each panel. Clinical details are summarized in Table 1. PPARγ positive cells (arrows) are detected in the germinative, periventricular, areas and in ependyma (double arrow) in cases, but not in controls. Insets show the localization of the optical field within the brain sections (arrowheads). Note the nuclear localization of PPARγ (A-G), the presence of PPARγ positive cell islets surrounding one IE positive cell in two fields from serial sections (F) and clusters of PPARγ immunoreactive cells around lesional tissue (G). Scale bar: 50 μm.

Gestational ages ranged from 23 weeks to 28 weeks, for cases and controls, so that all case samples could be compared with gestational age-matched controls. We first explored the level of infection in each sample by determining the total number of HCMV-positive cells in each slide, using an antibody specific to IE (Table 1, S2 Fig). No correlation could be established between the number of infectious foci and either gestational age, gender, or severity of the phenotype. Immunohistological analysis of PPARγ expression revealed PPARγ immunoreactive cells in the cell-dense, periventricular, brain germinative zone (BGZ) of all HCMV cases (Fig 9A–9E), but in none of the controls (Fig 8H and 8I). PPARγ staining was nuclear in the majority of cells (Fig 9A–9E), suggesting the presence of the active form of the receptor. We were also able to detect IE-positive cells surrounded by PPARγ positive cells (Fig 9F), supporting the hypothesis that viral replication enhances PPARγ expression both in host and neighboring cells. Isolated islets of PPARγ-positive cells were also detected in discrete lesional areas in the BGZ (Fig 9G). PPARγ-positive cells were also detected in the ependyma of HCMV subjects (Fig 9D and 9E).

**Fig 10:**
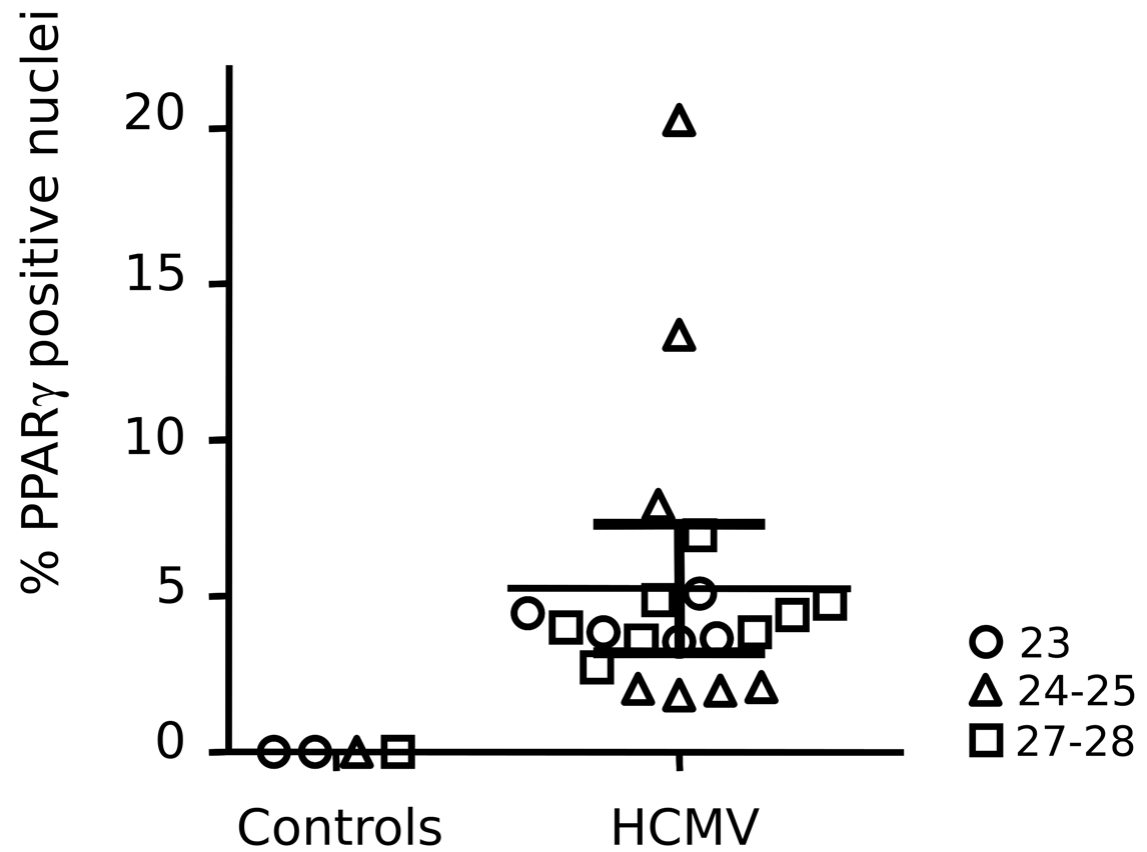
Summary of the immunohistological exploration of PPARγ expression in HCMV fetal cases and controls. Each symbol represents the mean relative numbers of PPARγ immunoreactive nuclei in optical fields (n = 6, magnification: x40) found in the brain germinal zone for each individual. Thin horizontal line indicates the average ratio of PPARγ positive cells found in patients, and thick horizontal lines indicate the corresponding SEM. Symbols indicate gestational age in weeks.

To assess the relative abundance of cells expressing PPARγ, we counted, for each case, the number of PPARγ positive nuclei in a series (n = 6) of optical fields within the BGZ (Fig 10). The field size was approximatively 10 mm^2^. The number of nuclei in the fields ranged from 453 to 1592, with an average value of 971.3 and a 5% confidence interval of 48.7. Cases showed individual variability in the abundance of PPARγ expressing cells, ranging from 1.82% to 20.28%, with a mean value of 5.25%. No correlation with gestational age was apparent.

**Fig 11:**
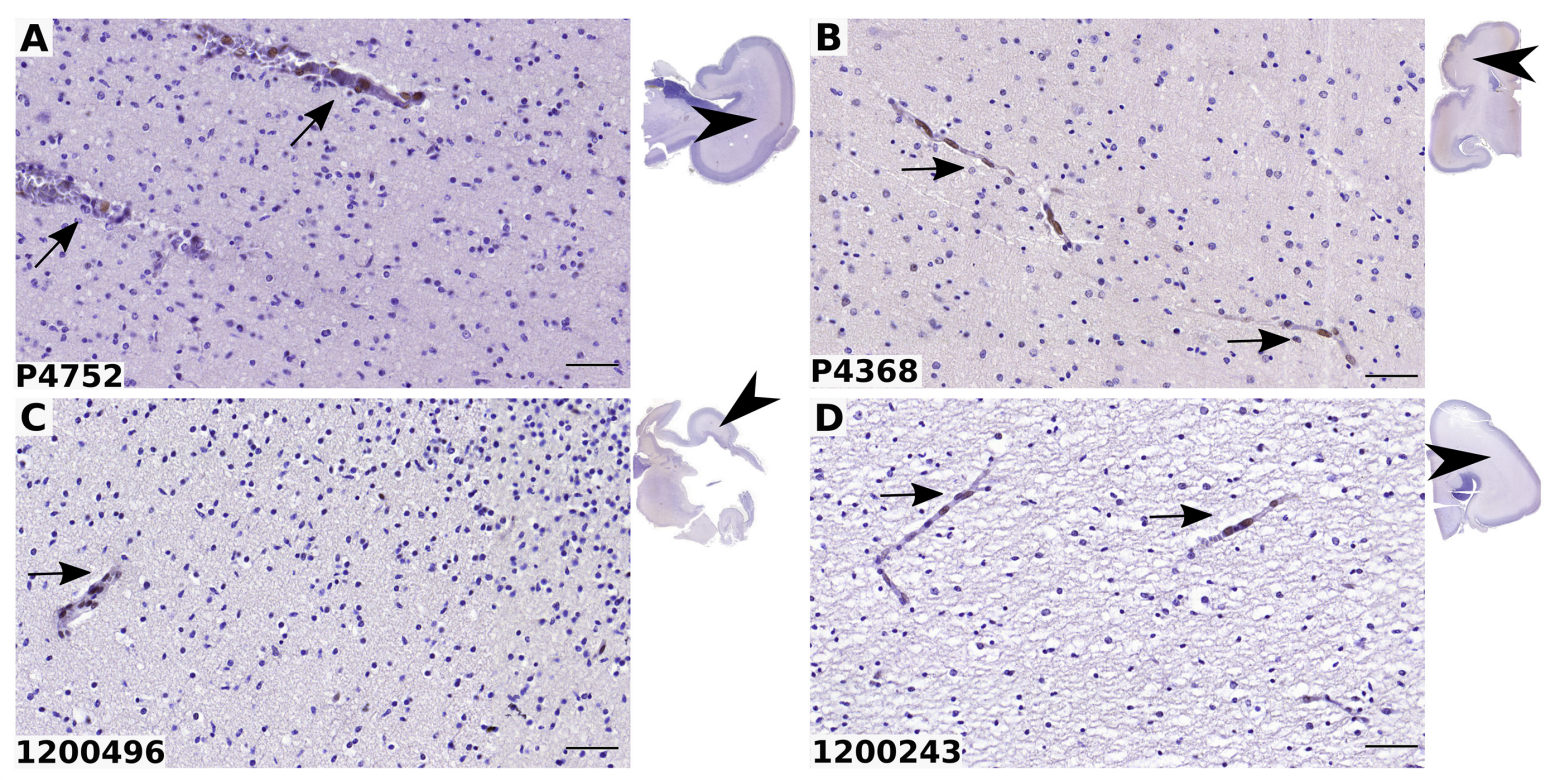
PPARγ expression is not detected in the white matter of HCMV-infected fetal brains. Shown are representative results of immunohistological staining of brain sections from fetuses infected by HCMV (A, B) or from controls (C, D). The reference number of each donor is indicated at the bottom left of each panel. Clinical details are summarized in Table 1. PPARγ positive cells (arrows) are detected in the vessels but not in the white matter in patients and controls. Insets show the localization of the optical field within the brain sections (arrowheads). Scale bar: 50 μm.

White matter was negative in all infected and control samples (Fig 11). PPARγ is physiologically expressed in vascular cells and is critical in vascular biology [27]. Accordingly, we observed that endothelial cells in brain vessels were positive to PPARγ in all infected and control samples (Fig 11). Together, these findings disclose that PPARγ expression is triggered specifically in the brain germinative areas of cases with congenital HCMV infection.

## Discussion

**Fig 12:**
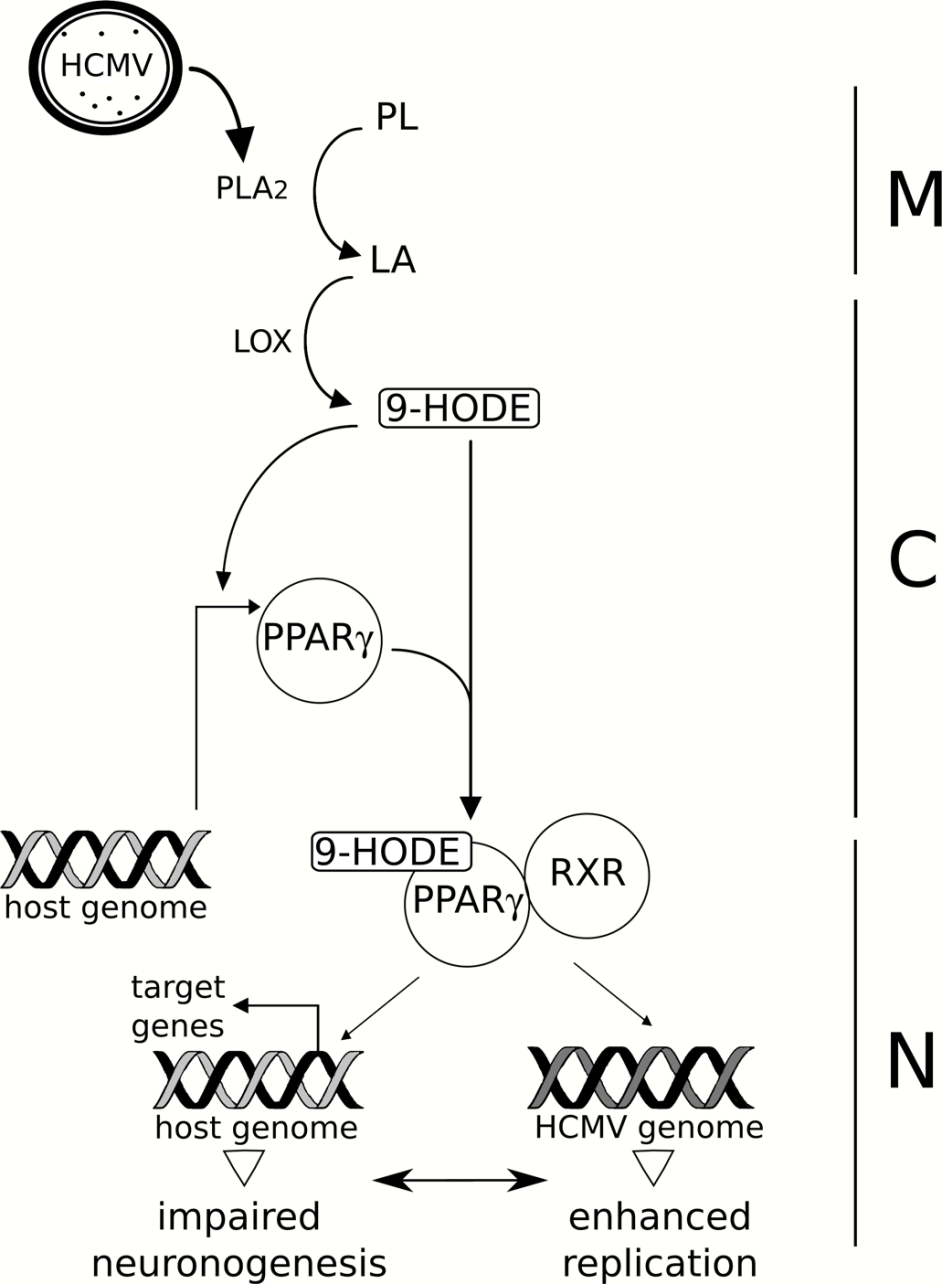
Proposed model for the role of PPARγ during HCMV infection of NSCs. HCMV particles (HCMV) carry onboarded a cell-derived packaged cPLA2 (oPLA2, dots), which catalyzes the release of linoleic acid (LA) from host membrane phospholipids (PL) upon infection. LA undergoes oxidization driven by 15-lipoxygenase (LOX), which generates 9-HODE. 9-HODE, in turn, increases PPARγ levels. Activated PPARγ dimerizes with RXR to regulate the expression of host and viral genomes, resulting in impaired neuronogenesis in vitro and enhanced viral replication. M: cell membrane, C: cytoplasm, N: nucleus.

The main result of our study is the identification of PPARγ activation as a molecular determinant of the pathology induced by HCMV infection in neural precursors, in vitro and presumably in vivo. Our findings unambiguously demonstrate that HCMV infection causes increased PPARγ levels and activity, increased biosynthesis of 9-HODE, impaired neuronogenesis and enhanced viral replication in NSCs (Fig 12).

We here showed that HCMV infection of NSCs associates with increased levels of PPARγ mRNA and protein, although the changes in levels of transcripts appeared much greater than that of the protein. It is now well documented that protein levels are not systematically proportional to mRNA levels [28–30]. The differences in mRNA and protein levels likely arise from the fact that Q-PCR relates to the steady-state levels of transcripts, but does not take into account mRNA stability, protein stability, or translation efficiency.

Enhanced activity of PPARγ appears to be a common feature of HCMV infection, both in NSCs and cytotrophoblasts [26]. In contrast, PPARγ activity is decreased in mouse lung tissues infected by H1N1 influenza A virus [31], whereas it increases in macrophages infected by *Mycobacterium tuberculosis* or *Mycobacterium bovis* [32]. The observation that 9-HODE stimulation of uninfected NSCs is sufficient to increase PPARγ levels agrees with previous studies which revealed that 9-HODE selectively increases PPAR*γ* gene expression in human U937 monocytic cells [33] or mesangial cells [34]. We have recently reported that 13-HODE and 15-HETE were the PPARγ agonists secreted by cytotrophoblasts and placenta explants infected by HCMV. In this case, however, no 9-HODE changes were detected [24]. This suggests tissue specificity in the response to HCMV infection with regard to fatty acids metabolism. Strikingly, 9-HODE, 13-HODE and 15-HETE all arise from oxidization of linoleic (9/13-HODE) or arachidonic (15-HETE) acids by lipoxygenase 15 [16]. This route of biosynthesis of PPARγ activating lipids is alternate to that previously described in human foreskin fibroblasts, where cyclooxygenase 2 activity catalyzes the biosynthesis of 15d-PGJ_2_ from arachidonic acid [35]. Some NSCs showed activated PPARγ and no IE detectable expression in infected monolayers (Fig 4A). We showed that infected cells exert a bystander effect on PPARγ expression in uninfected cells through soluble mediators. A significantly increased production of the highly membrane-permeant 9-HODE in infected cells could explain such a bystander effect, although we were unable to detect increased amounts of 9-HODE in the supernatants. One likely hypothesis is that 9-HODE could be present at detectable amounts only in live infected cells and that it would be released upon lysis of infected cells.

Abnormal PPARγ activity is likely to have multiple outcomes in infected cells and appears as an efficient strategy for HCMV to target simultaneously a number of important functions in the host NSC. PPARγ is required for IE gene expression and efficient HCMV replication in the host cell [26]. It exerts neuroprotective and anti-inflammatory effects, and regulates the oxidative pathway [36–39]. In particular, PPARγ is able to trans-repress the activity of NF-κB, AP-1 and STAT-1 as a response to their activation in the infectious context, resulting in negative modulation of production of the inflammatory mediators iNOS, TNFα, and IL-6 [37]. We report defective neuronal differentiation of NSCs infected by HCMV, in agreement with previous investigations [8, 10, 11, 13, 14]. Previous studies reported that PPARγ agonists either inhibited [40, 41], promoted [42, 43] or had no effect [44] on neuronal differentiation of uninfected rat or mouse neural progenitors. Our findings, most particularly our assays with singled-out expression of PPARγ in NSCs or using 9-HODE or T0070907, demonstrate unambiguously that PPARγ activity inhibits neuronogenesis in NSCs. To rule out the possibility of a receptor-independent effect of T0070907, it would be necessary to knockdown PPARγ expression in NSCs. Unfortunately, we failed to obtain any viable PPARγ knockdown using specific siRNA or shRNA vectors. Future experiments using CRISPR/CAS-9 system to invalidate PPARγ expression in NSCs may help elucidate the role of PPARγ in neurogenic differentiation.

In the present study, NSCs were generated through early neuroepithelial differentiation of human ES cells by using inhibitors of the TGFβ superfamily and a defined medium containing N2 and B27 supplements [19]. This method allows for efficient neural commitment and avoids possible confounding factors such as donor variability, batch-dependent components and feeder cell conditioned media. NSCs displayed a cortical phenotype and no immunoreactivity to non cortical markers [19], which is likely critical since HCMV infection targets cortical areas of the developing brain [7]. A recent study investigated HCMV replication in primitive prerosette NSCs (pNSCs), which represent a very primitive neural developmental stage [45]. That study revealed that viral replication depends on the differentiation status of the target cells. In that study, neither expression of HCMV early antigens or viral spreading could be evidenced from pNSCs or derived progenitors. We assume that the NSCs used in our work represent a later differentiation stage than the progenitors used in that previous study. pNSCs do not express the radial glia progenitor cell marker GFAP and can be differentiated to S100β^+^ NPCs following treatment with FGF2 [45]. This may explain why our NSCs readily supported viral replication, exhibited strong expression of early and late antigens, showed assembled viral particles, and allowed for efficient spreading. Further studies are required to decipher why the initial stages of infection were delayed, with only a minor part of the NSC population becoming IE-positive during the two first days. The reasons for the delayed initial kinetics of infection are unclear, but may be related to either changes in the surrounding cells resulting in increased permissivity, or by possible changes in the virus cycle. Notably, it is presently unclear whether the number of morphologically mature virions detected in infected NSCs by transmission electron microscopy fully correlates with infectivity (Fig 2).

The involvement of PPARγ in the pathogenesis of congenital HCMV infection is strongly supported by its pattern of expression in HCMV-infected human brain samples. Indeed, all samples from HCMV subjects, and none from control cases, showed PPARγ immunostaining in the brain germinative zone (BGZ). Moreover, BGZ appeared to be the only brain area with detectable abnormal PPARγ expression in infected subjects, unlike blood vessels or white matter. So far, only the presence of inclusion bodies [2], but not that of any specific protein, has been reported in brain sections from cases congenitally infected by HCMV. Our findings are consistent with studies in the mouse which identified the germinative subventricular zone as the most sensitive site to infection by murine cytomegalovirus (MCMV) [7, 46]. The critical role of PPARγ in neurodevelopmental regulation requires fine spatiotemporal tuning of expression and activity [47]. Therefore, we assume that asynchronously increased PPARγ activity could be deleterious to neurogenesis during HCMV congenital infection. Notably, we observed that the number of PPARγ expressing cells in brains slices from an infected case was always similar or greater than the number of IE positive cells. This is consistent with the possibility of a bystander effect from infected cells to uninfected cells during congenital infection, such as that observed in vitro with NSCs.

In conclusion, NSCs turned out to be an invaluable tool for modeling functional correlates of HCMV infection, and this cell platform may probably be extended to other viral pathologies of the central nervous system. Our findings shed a new light on the pathophysiological bases of the neurological outcomes of congenital HCMV infection and on the role PPARγ in neural stem cell and developing brain.

## Materials and Methods

### Ethics statement

Neural stem cells from human embryonic stem cells were used in the frame of a project approved by the French authorities (Agence de la Biomedecine, authorization number SASB0920178S). Collection of brain histological samples was performed in the frame of a project coordinated by Necker Hospital, AP-HP (Assistance Publique-Hôpitaux de Paris). The study was performed in accordance with the French ethical guidelines and was approved by the French authorities (Agence de la Biomedecine, authorization number PFS-15009). Written informed consent was obtained from all study participants prior to sample collection. All samples were anonymized before processing.

### Cells, viruses and reagents

NSCs were generated and grown in vitro as detailed elsewhere [18]. Briefly, neural tube-like structures containing neuro-epithelial cells were obtained in vitro from human embryonic stem (ES) cells by using two inhibitors of the TGFβ superfamily (SB431542 and Noggin). Manually isolated colonies were expanded in the presence of EGF, FGF2 and BDNF to obtain NSCs. NSCs were seeded at 100 000 cells/cm^2^ and maintained in growth medium (basal medium) consisting of DMEM/F12/Neurobasal medium (Life Technologies, Grand Island, NY, USA) mixed at a ratio of 1/1/2 (v/v/v) in the presence of N2 and B27 supplements (Life Technologies), 10 ng/ml FGF2, 10 ng/ml EGF, 20 ng/ml BDNF (all from Peprotech, Rocky Hill, NJ, USA). Culture supports were coated first by PBS containing 0.05% poly-ornithine (PO) (Sigma, Saint-Louis, MO, USA) then by PBS containing mouse laminin (1 μg/cm^2^)(Roche, Basel, Switzerland). NSC cultures were checked for the absence of mycoplasma (Plasmotest, Invivogen, Toulouse, France). Neuronogenic differentiation was induced by removal of FGF2 and EGF and addition of laminin (2 μg/cm^2^) into the medium (differentiation medium) of low passage (<12) NSCs seeded at 50 000 cells/cm^2^. Culture medium was renewed every two days.

The human immortalized fibroblast line MRC5 (ATCC CCL171, Manassas, VA, USA) was cultured in DMEM containing 10% bovine calf serum (Life technologies).

We used the clinical VHL/E HCMV strain (a gift from C. Sinzger, Tubingen, Germany), at low passage (<8) of amplification in MRC5 cells, and laboratory-adapted AD169 HCMV strain (ATCC VR538). Virus stocks were collected from infected MRC5 fibroblasts when cytopathic effects were >90%. Supernatants were clarified of cell debris by centrifugation at 1,500 × *g* for 10 min, ultracentrifuged at 100,000 × *g* for 30 min at 4°C, harvested in NSC basal medium, and stored at −70°C until use. Virus titers were determined upon infection of MRC5 cells by serial dilutions of the inoculum followed by immunofluorescence analysis to count the number of nuclei immunoreactive to HCMV Immediate Early antigen (IE) 24 h post infection (pi)(fluorescence unit forming assay). UV irradiation of HCMV particles was performed for 30 min in a closed propylene tube with a Spectrolyne irradiator (EF-140/F) fitted with a BLE-2537S bulb [254 nm] (Spectronics corporation, Westbury, NY). In these conditions, the theoretical radiant energy density is 36 J/cm^2^. After such a treatment, irradiated HCMV virions were still able to infect cells 30 min pi, as checked by immunostaining with an antibody specific to the tegument protein pp65 (Virusys corporation, Taneytown, MD), but the viral genome could not be expressed 24 h pi, as checked by the absence of immunoreactivity to the HCMV Immediate Early antigen (IE).

Antibodies specific to PPARγ were H-100 (Santa Cruz Biotechnology, Dallas, TX, USA) and A3409A (Abcam, Cambridge, UK). We used primary antibodies specific to HCMV IE (Argene, Verniolle, France), PPARα (H-74, Santa Cruz Biotechnology), PPARβ (H-98, Santa Cruz Biotechnology), RXRα, β and γ (DN197, Santa Cruz Biotechnology), SOX2 (D6D12, Cell Signaling Technology, Beverly, MA, USA), HUC/D (16A11, Life Technologies), Nestin (10c2, Millipore, Billerica, MA, USA), Class III beta-tubulin (βIII tubulin) (TU-20 or ab18207, both from Abcam), cleaved caspase-3 (Cell Signaling Technology), Ki67 (KiS5, Millipore). Secondary antibodies against rabbit or mouse immunoglobulins were conjugated with Alexa-488 or -555 or -647 fluorophores (Life Technologies). No staining was detectable when cells were incubated with the secondary antibodies alone, or with primary and secondary antibodies for which species of origin did not match.

PPARβ synthetic activator was rosiglitazone (1 μM) (Sigma) and a stimulation time of 2 h was used. PPARβ specific inhibitor was T0070907 (10 nM) (Sigma). Optimal concentrations of rosiglitazone and T0070907 were determined from initial dose-effect experiments using a luciferase PPAR reporter plasmid (as detailed below). Control experiments with rosiglitazone were performed with the vehicle, DMSO. Synthetic 9-HODE was purchased (Cayman, Ann Harbor, MI). Control experiments with synthetic 9-HODE were performed with its vehicle, ethanol.

Cell-derived, virion-packaged cPLA_2_ was inactivated as described elsewhere [24]. Viral suspensions were incubated in a volume of 1 ml for 1 h at room temperature in the presence of 50 μM methyl arachidonyl fluorophosphonate (MAFP; Sigma), ultracentrifuged at 100,000 × *g* for 30 min at 4°C, washed twice with phosphate-buffered saline (PBS; Life Technologies) and diluted in culture medium. The working concentration of MAFP (50 μM) was determined by immunofluorescence analysis using NSCs infected by HCMV particles treated by various doses of MAFP, using an antibody specific to IE. Cell viability was checked by 4′6-diamino-2-phenylindole (DAPI) staining. Control viral suspensions were processed identically after incubation in the presence of the vehicle (DMSO) instead of MAFP. When NSCs were infected with MAFP-treated virus, control uninfected NSCs were cultured in the presence of MAFP at a concentration equivalent to that which would have been obtained without the HCMV particles washes (50 nM).

### Transmission electron microscopy

NSC cultures infected by HCMV at a MOI of 10 were fixed in 2% glutaraldehyde in 0.1 M Sorensen phosphate buffer (pH 7.4) for 4 h at 4°C, 6 days post infection. After an overnight wash in 0.2 M phosphate buffer, the cells were post-fixed for 1 h at room temperature with 1% osmium tetroxide in 250 mM saccharose and 0.05 M phosphate buffer, and stained overnight in 2% uranyle acetate. The samples were then dehydrated in a series of graded ethanol solutions and embedded in an Epon-Araldite resin (Embed 812-Araldite 502, Electron Microscopy Sciences, Hatfield, PA). Finally, the cells were sliced into 70-nm thick sections and mounted on 200-mesh collodion-coated copper grids prior to staining with 3% uranyle acetate in 50% ethanol and Reynold’s lead citrate. Examinations were carried out on a transmission Hitachi HU12A electron microscope at an accelerating voltage of 75 kV.

### Western blot

NSC cultures were seeded in 10 cm^2^ dishes at a density of 100, 000 cells/cm^2^. NSCs were infected (MOI 10) or stimulated 16 h after plating, lysed in RIPA buffer containing 50 mM Tris-HCL, pH 7.6; 150 mM NaCl; 0.1% sodium deoxycholate, 0.1% sodium dodecylsulfate, 0.1% NP-40, 1 mM EDTA, and a protease inhibitor cocktail (all from Sigma). Lysates were subjected to SDS-PAGE with 4 to 12% Tris-Tricine gels (Life Technologies). Proteins were blotted onto nitrocellulose membranes (GE-Healthcare, Pittsburgh, PA, USA) using a semi-dry transfer device (Biorad, Hercules, CA, USA). Western blot was performed using Tris-Buffered-Saline (TBS) containing 0.1% Tween-20 as the wash buffer, TBS containing 5% non-fat dry milk and 3% BSA as the blocking buffer, and primary or horseradish peroxidase-conjugated secondary antibodies diluted in blocking buffer. Detection was carried out by using a chemoluminescence kit (Sigma). Analysis was performed with a Chemidoc system (Bio-Rad, Hercules, CA) using conditions when signals were not saturating.

### Immunofluorescence

Cells were cultured on coverslips coated with PO and laminin, fixed in 100% methanol for 15 min at -20°C (PPAR staining), or in 4% formaldehyde for 20 min at 4°C, and permeabilized in 0.3% Triton X-100 for 15 min at room temperature (other stainings). Blocking buffer was PBS containing 5% fetal calf serum. Primary antibodies were diluted in blocking buffer and applied overnight at 4°C. Secondary antibodies were diluted in blocking buffer and applied for 1 h at room temperature. The cells were washed three times with blocking buffer, then washed three times with PBS, then counterstained with 1 μg/ml DAPI (Sigma), washed again three times with PBS and visualized on an DM4000B inverted fluorescence microscope (Leica, Solms, Germany). Image processing was performed using ImageJ software [48].

### In vitro neurogenesis assays

For neuronogenesis assays, NSCs were seeded in 0.35 cm^2^ coated culture wells at a density of 15,000 cells per well in differentiation medium and were infected 24 h later. Next, immunofluorescence was carried out at different time points pi, using double staining with the antibodies specific to HUC/D and SOX2, and counterstaining with DAPI. The culture plates were analyzed with an automated microscopy device (Cellomics) to count the number of nuclei positive for SOX2 or HUC/D. DAPI staining was used as the primary mask. Cell death assays were performed similarly, using the reagent Image-it dead (Life Technologies).

For neuronogenesis assay in the presence of T0070907, NSCs were installed in differentiation medium on 15 mm^2^ coated glass coverslips in 2.3 cm^2^ wells (150,000 cells/well). The day after, NSCs were infected or not by HCMV at a MOI of 1. T0070907 (10 nM) was added to the medium 3 h after infection. The medium was renewed everyday. At day 4 pi, immunofluorescence analysis was performed as described above using antibodies specific to HUC/D, SOX2, or IE, and DAPI as a counterstain. Twelve optical fields of each coverslip were visualized on a DM4000B inverted fluorescence microscope, and analyzed using ImageJ. The number of cells immunoreactive to SOX2, HUC/D or IE antibodies was counted manually to exclude dead cells and to resolve cell clusters. Three independent experiments were performed.

### Luciferase reporter assays

We used a firefly luciferase (Luc) reporter plasmid based on a pGL4 backbone (Promega, Madison, WI, USA) containing three PPAR responsive elements (PPREs) [26] upstream of the herpes simplex thymidine kinase promoter (pGL4-PPRE-luc). For normalization, we used a promoter-less renilla luciferase normalization plasmid (pRL-null, Promega). NSCs were seeded in 96-well plates at a density of 25,000 cells per well. Transfection of both the reporter and normalization plasmids was performed 16 h after seeding using Genejuice transfection reagent (Millipore), according to the manufacturer's instructions. Cells were infected (MOI 10) or treated with rosiglitazone (1 μM) or T0070907 (10 nM) during the 24 h following transfection. Last, cell lysis was performed using Cell Culture Lysis Reagent (Promega). Luciferase activity was quantified using a Centro luminometer (Berthold). All assays were done in triplicate and the experiment was repeated twice.

### High performance liquid chromatography coupled to tandem mass spectrometry (LC-MS/MS)

LC-MS/MS was performed as detailed elsewhere [26], using HPLC grade methanol, methyl formate, and acetonitrile (Sigma–Aldrich). Deuterium-labeled lipoxin A4 (LxA4-d5), leukotriene B4 (LTB4-d4) and 5- hydroxyeicosatetraenoic acid (5-HETE-d8) (Cayman Chemicals) were mixed at a concentration of 400 ng/ml in methanol and used as the internal standard (IS) solution. In all experiments, NSCs from 10 cm^2^ culture wells were harvested 6 h pi in 0.2 ml of PBS, transferred to lysing matrix A (MP Biomedicals) and supplemented with 5 μl of IS solution.

Cells were lysed using a spin homogeneizer (Fastprep, MP Biomedicals) with 2 cycles of 20 sec at 5,000 rpm. 10 μl of the lysed cell suspension were added to 200 μl of 0.1 M NaOH for subsequent protein quantification using a Bradford assay (BioRad). The remaining of the lysate was supplemented with 200 μl methanol, vigorously shaken, and centrifuged for 15 min at 1,000 x *g* at 4°C. The supernatants were collected and stored at -80°C until lipid extraction. Lipid amounts from cell lysates were expressed in pg per mg of protein in the lysate. Culture supernatants were collected 6 h pi, supplemented with 300 μl of ice-cold methanol and 5 μl of IS solution, clarified by a centrifugation at 1,000 x *g* for 15 min, and stored at -80°C until lipid extraction. Lipid amounts from supernatants were expressed as pg/ml.

Lipid preparation from all samples was carried out through solid-phase extraction using hydrophobic polystyrene-divinylbenzene resin in dedicated 96-well plates (Chromabond multi96 HR-X 50 mg; Macherey-Nagel). After conditioning of the plate with methanol and sample loading, the plates were washed twice with H_2_O/MeOH (90/10, v/v) and dried under aspiration for 15 min. Lipids were eluted with methanol (2 ml), dried under nitrogen, dissolved again in methanol (10 μl) and transferred to liquid chromatography tubes before LC–MS/MS analysis.

LC-MS/MS analysis was performed using an UHPLC system (LC1290 Infinity, Agilent) coupled to a 6460 triple quadrupole mass spectrophotometer (Agilent Technologies) fitted with an electro-spray ionization interface. Separation was done at 40°C on a Zorbax SB-C18 column (2.1 mm–50 mm–1.8 μm) (Agilent Technologies). The compositions of mobile phases A and B were water, acetonitrile (ACN) and formic acid (FA) (75/25/0.1) and ACN, FA (100/0.1), respectively. Compounds were separated with a linear gradient from 0 to 85% B in 8.5 min and then to 100% B at 9 min. Isocratic elution continued for 1 min at 100% B, then 100% A was reached at 10.2 min and maintained to 11 min. The flow rate was 0.35 ml/min. The autosampler was set at 5°C and the injection volume was 5 μl. Source conditions were as follows: negative ESI mode, source temperature = 325°C, nebulizer gas (nitrogen) flow rate = 10 l/min, sheath gas (nitrogen) flow rate = 12 l/min, sheath gas temperature = 400°C and spray voltage = −3500 V. Data were acquired in MRM mode. For each compound, the best conditions of separation and quantification were defined: retention time in minutes (RT), specific Q1/Q3 transition (T) fragmentor (F) and collision energy (CE). Peak detection, integration and quantitative analyses were performed using Mass Hunter Quantitative analysis software (Agilent Technologies). At least three independent experiments were performed, each in triplicate wells.

### Generation of recombinant lentiviral vectors and NSCs

We used pWPXL-GFP (Addgene #12257), a lentiviral vector backbone allowing for stable expression of enhanced Green Fluorescent Protein (eGFP) driven by the human EF1α gene promoter. A MluI- XbaI fragment containing the wild type mouse *Pparg*2 cDNA (1626 bp) was excised from a modified pSV Sport PPARγ2 plasmid (Addgene #8862) [49] and substituted to the eGFP cDNA into pWPXL-GFP restricted by MluI and SpeI (plasmid pWPXL-Pg). A puromycin resistance cassette containing the gene *Pac* under the control of the human ubiquitin promoter was excised by AscI digestion of the plasmid pSF-CMV-Ub-Puro-SV40 Ori SbfI (Oxford genetics), blunted, and inserted into the blunted KpnI site of pWPXL-Pg and pWPXL-GFP, generating the plasmids plenti-Pg and plenti-GFP.

Lentiviral vectors were generated by transfection of the plasmids pMD2G (Addgene #12259), pCMVR8.74 (Addgene #22036), and plenti-GFP or plenti-Pg into HEK293 cells using calcium phosphate, as recommended by the supplier (Clontech). Lentiviral particles were collected at 24 h and 48 h post transfection, ultracentrifuged at 50,000 x *g* for 120 min at 16°C, resuspended in NSC basal medium, and stored at −70°C until use. Recombinant NSCs were generated by transducing cultures by the lentiviral vectors, followed by continuous selection by 1 μg/ml puromycin. Ectopic expression of eGFP or PPARγ was checked by immunofluorescence, western blot and oil red O staining.

### Oil Red O staining

Uninfected or infected (MOI 10) cells were incubated for 2 h in growth medium in the presence of oleic acid conjugated with BSA (10 μg/ml), fixed and permeabilized in methanol for 2 min at −20°C and incubated for 10 min with 0.3% Oil Red O diluted in 60% isopropanol. After 30 s of incubation, cells were washed in water and nuclei were counterstained with hematoxylin.

### Quantitative RT-PCR

RNA was extracted by using dedicated columns (Qiagen), and 1 μg was reverse transcribed (RT) with Superscript III (Invitrogen), according to the supplier’s recommendations. All quantitative RT-PCR (Q-PCR) assays were based on a SyBr-green based PCR mixture (Roche) using a LC480 system (Roche). All primers pairs were designed using the Primer3 software (http://frodo.wi.mit.edu/) [50] and characterized by real-time amplification of a series of cDNA dilutions to determine linearity range and primer efficiency. Primer sequences are available upon request. All Q-PCR amplifications were done in triplicate and the experiments were performed at least twice. Q-PCRs were carried out according to the MIQE guidelines [51]. Reference gene was GAPDH, as identified by Genorm analysis.

### Chromatin immunoprecipitation

Chromatin immunoprecipitation (ChIP) was carried out using the Magnetic ChIP kit (Pierce) following the supplier’s recommendations. NSCs were seeded at a density of 100,000 cells / mm^2^ onto 60-mm plates containing one 12-mm glass coverslip, infected after 16 h at a MOI of 10 (AD169 strain), and fixed 48 h post infection. Infection was controlled by immunofluorescence analysis of the cells on the coverslip, using an antibody specific to IE. Sonication of chromatin was performed using a Vibracell device (Bioblock Scientific) and checked by agarose gel electrophoresis. Chromatin was immunoprecipitated with 10 μg of specific antibody or 10 μg of unspecific mouse immunoglobulins. One tenth of the immunoprecipitated DNA samples and 5 ng of input DNA samples were subjected to Q-PCR for normalization. Primers specific to *DLK1* were described elsewhere [20].

### Immunohistopathological analyses

Brain tissue biopsies were collected from 20 human fetuses aborted electively because of HCMV congenital infection and from 4 controls aborted for non-infectious diseases. Immuno-histopathological brain analysis of control and HCMV subjects was performed on 8 μm sections from paraffin blocks using standard methods, IE antibody, E8 PPARγ antibody, and Mayer’s hematoxilin counterstain, with a Dako Autostainer automated device (Dako, Glostrup, denmark). Slides were scanned with a Panoramic 250 system (3D Histech, Budapest, Hungary) and analyzed with the Panoramic viewer software (3D Histech). For each patient, 6 optical fields within the brain germinative zone were analyzed. The total number of nuclei in each field was determined using the Fast Morphology plug-in of ImageJ software, with a threshold size of 50 square pixels. When required, cell clusters were resolved manually. The number of nuclei with positive PPARγ staining in each field was determined manually to exclude endothelial cells when present.

### Statistical analysis

Statistical analyses were performed with the StatEL plugin (Adscience) for Excel (Microsoft, Redmond, WA) or GraphPad Prism (GraphPad Software, San Diego, CA), using Kruskall-Wallis test unless indicated. Error bars show 5% confidence intervals (CI).

### Accession numbers

PPARγ gene: *PPARG*, Ensembl ID: ENSG00000132170

Nestin gene: *NES*, Ensembl ID: ENSG00000132688

SOX2 gene: *SOX2*, Ensembl ID: ENSG00000181449

HUC/D gene: Ensembl ID: *ELAVL4*, ENSG00000162374

β3-Tubulin gene: *TUBB3*, Ensembl ID: ENSG00000258947

DLK1 gene: *DLK1*, Ensembl ID: ENSG00000185559

## Supporting Information

S1 Fig HCMV infection of growing NSC culture does not alter SOX2 expression. Representative immunofluorescence analysis of NSC cultured in proliferation medium and infected (HCMV, MOI 10) or not (NI) using antibodies against SOX2 and IE, and DAPI counterstaining, 48h post infection. Arrowheads indicate nuclei remnants from dead cells. Scale bar: 25 μm.
(TIF)

S2 Fig IE expression in brain germinative zones from fetus with congenital HCMV infection. Shown are representative results of immunohistological staining of brain sections from fetuses infected by HCMV (top row and bottom row, left) or from control (bottom row, right) using an antibody against IE. Arrowheads denote IE-positive cells. The reference number of each donor is indicated at the bottom right of each panel. Subjects 4752 and 4082 are representative of cases with numerous IE positive cells (top left); subject 4082 is representative of cases with rare IE positive cells (top right). Clinical details are summarized in Table 1. Magnification: x25. Scale bar: 100 μm.
(TIF)
