# Supplementary figures and images for: PPARγ Is Activated during Congenital Cytomegalovirus Infection and Inhibits Neuronogenesis from Human Neural Stem Cells
Source: PLoS Pathog. 2016 Apr 14;12(4):e1005547. doi: 10.1371/journal.ppat.1005547 (PMC4831785; doi:10.1371/journal.ppat.1005547)

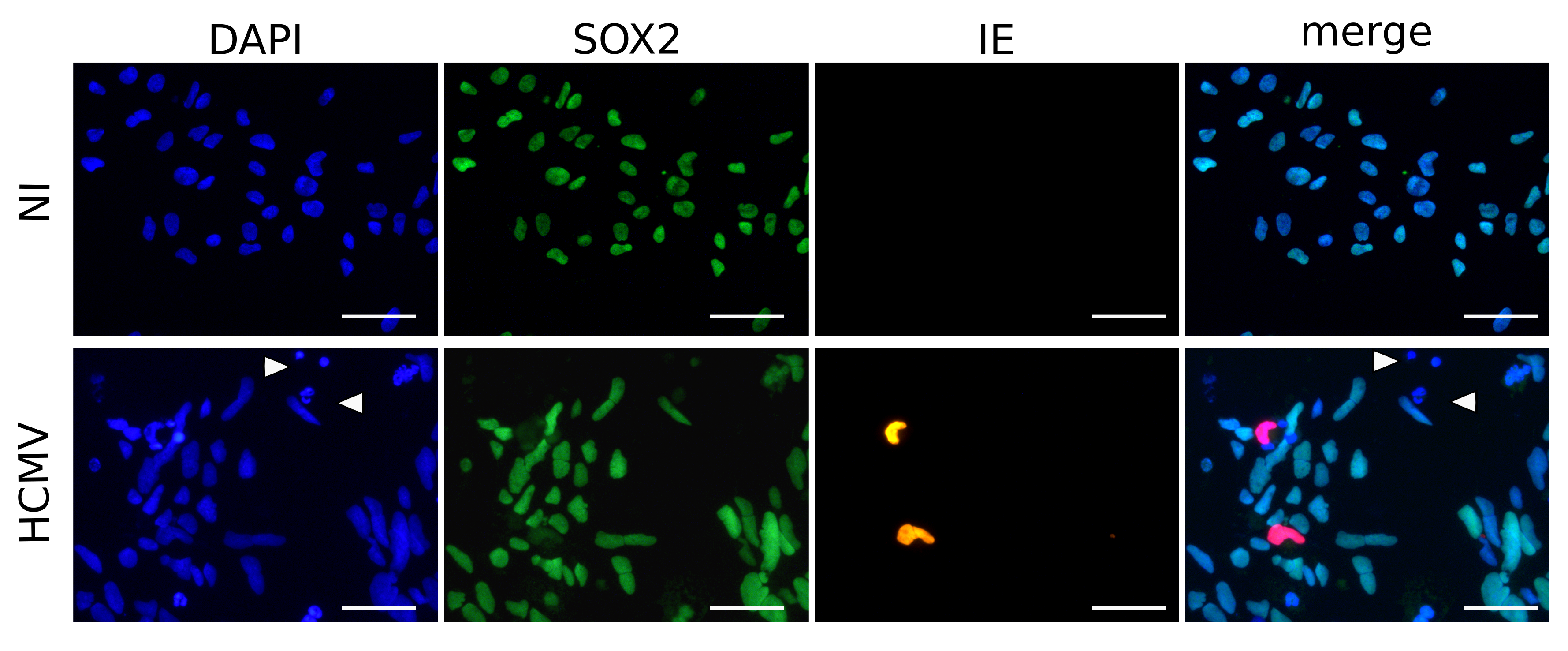

Supplement: S1 Fig — Representative immunofluorescence analysis of NSC cultured in proliferation medium and infected (HCMV, MOI 10) or not (NI) using antibodies against SOX2 and IE, and DAPI counterstaining, 48h post infection. Arrowheads indicate nuclei remnants from dead cells. Scale bar: 25 μm. (TIF) [file ppat.1005547.s001.tif]

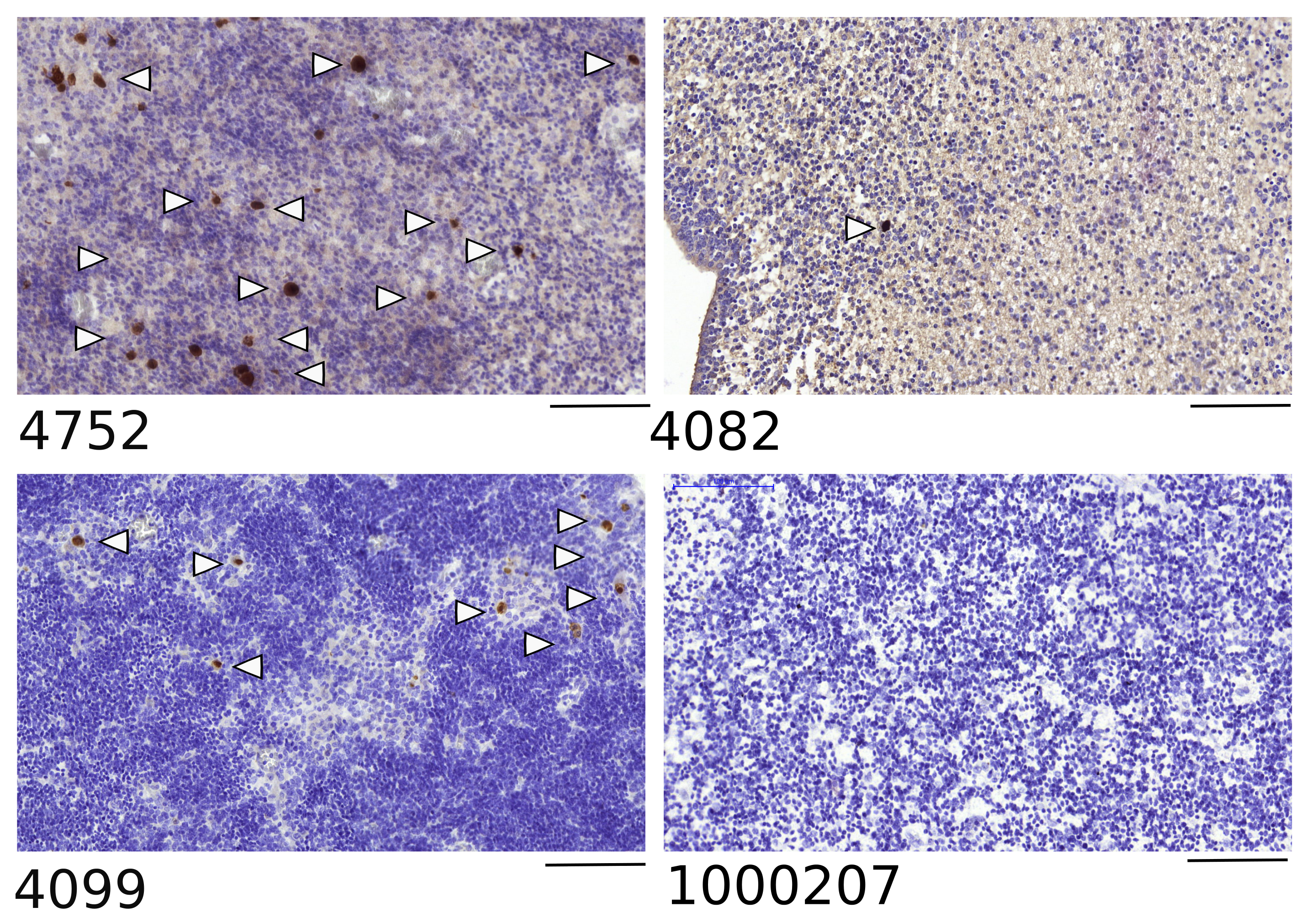

Supplement: S2 Fig — Shown are representative results of immunohistological staining of brain sections from fetuses infected by HCMV (top row and bottom row, left) or from control (bottom row, right) using an antibody against IE. Arrowheads denote IE-positive cells. The reference number of each donor is indicated at the bottom right of each panel. Subjects 4752 and 4082 are representative of cases with numerous IE positive cells (top left); subject 4082 is representative of cases with rare IE positive cells (top right). Clinical details are summarized in Table 1. Magnification: x25. Scale bar: 100 μm. (TIF) [file ppat.1005547.s002.tif]
